# An Innovative Approach for The Integration of Proteomics and Metabolomics Data In Severe Septic Shock Patients Stratified for Mortality

**DOI:** 10.1038/s41598-018-25035-1

**Published:** 2018-04-27

**Authors:** Alice Cambiaghi, Ramón Díaz, Julia Bauzá Martinez, Antonia Odena, Laura Brunelli, Pietro Caironi, Serge Masson, Giuseppe Baselli, Giuseppe Ristagno, Luciano Gattinoni, Eliandre de Oliveira, Roberta Pastorelli, Manuela Ferrario

**Affiliations:** 1 Politecnico di Milano, Milan, Italy; 2 Proteomics Platform - Parc Científic de Barcelona, Barcelona, Spain; 3 IRCCS-Istituto di Ricerche Farmacologiche Mario Negri, Milan, Italy; 4 Anestesia e Rianimazione, Azienda Ospedaliero-Universitaria S. Luigi Gonzaga, Orbassano, Italy; 5 Dipartimento di Oncologia, Università degli Studi di Torino, Turin, Italy; 6 Department of Anesthesiology, Emergency and Intensive Care Medicine, University of Göttingen, Göttingen, Germany

## Abstract

In this work, we examined plasma metabolome, proteome and clinical features in patients with severe septic shock enrolled in the multicenter ALBIOS study. The objective was to identify changes in the levels of metabolites involved in septic shock progression and to integrate this information with the variation occurring in proteins and clinical data. Mass spectrometry-based targeted metabolomics and untargeted proteomics allowed us to quantify absolute metabolites concentration and relative proteins abundance. We computed the ratio D7/D1 to take into account their variation from day 1 (D1) to day 7 (D7) after shock diagnosis. Patients were divided into two groups according to 28-day mortality. Three different elastic net logistic regression models were built: one on metabolites only, one on metabolites and proteins and one to integrate metabolomics and proteomics data with clinical parameters. Linear discriminant analysis and Partial least squares Discriminant Analysis were also implemented. All the obtained models correctly classified the observations in the testing set. By looking at the variable importance (VIP) and the selected features, the integration of metabolomics with proteomics data showed the importance of circulating lipids and coagulation cascade in septic shock progression, thus capturing a further layer of biological information complementary to metabolomics information.

## Introduction

In the last decade, advances in high-throughput proteomic and metabolomic techniques allowed to evaluate the association of genetic and phenotypic variability with disease progression. This aspect is fundamental in case of complex multifactorial syndromes, such as sepsis and septic shock, which are both characterized by a response to treatment different from patient to patient and extremely difficult to predict. Moreover, the same root cause (e.g. source of primary infection or pathogen) may lead to significantly different clinical phenotypes and outcomes.

In recent years, the number of software aiming at integrating multiomics data is increasing^1^. The term “multi-omics data integration” refers to a new scientific request of combining multiple sources of information (omics) to provide deeper biological understanding. However, the majority of them are mainly devoted for interpreting genomics data and are based on a mechanistic interpretation, as the network-based (NB) approaches that take into account currently known (e.g. protein-protein interactions) or predicted (e.g. from correlation analysis) relationships between biological variables^1^. In this class, graph measures (e.g. degree, connectivity, centrality) and graph algorithms (e.g. sub-network identification) are used to identify valuable biological information. A different approach is based on statistical methods in order to find possible associations among variables and outcomes. Many softwares based on mathematical approaches usually adopted for data-mining or machine learning, are now freely available^2^. They are developed in Matlab^TM^ or R environment and perform specific analyses on data given as input without any further constrains^3,4^. The availability of such software has encouraged this field, but has not solved the problem how to deal with different types of data (e.g. different standards and different normalization approaches) and how to deal with a low number of observations, highly correlated variables, cross-over or longitudinal study. Different type of dataset requires different approaches and strategies. The comparison of the results from different models represents a valuable approach to support any possible associations found among variables.

In this study, we examined plasma metabolome, proteome and clinical features in a subset of patients with the most severe manifestation of sepsis, enrolled in the multicenter, randomized clinical trial ALBIOS (Albumin Italian Outcome Sepsis study, NCT00707122^5^). Patients were divided into survivors (S) and non-survivors (NS) according to 28-day mortality and plasma samples were collected one day (D1) and one week (D7) after diagnosis of septic shock. Integration of metabolomics and proteomics information was aimed at revealing molecular pathways as well as at identifying molecular features decisive in stratifying the patients.

In our previous study^6^ we found that profiles of specific metabolites, measured separately at D1 and at D7, markedly differed between survivor and non-survivor patients. More precisely, we observed that low unsaturated long-chain phosphatidylcholines (PCs) and lysophosphatidylcholines (lysoPCs) species were associated with mortality together with circulating kynurenine. Therefore, we speculated that lipid homeostasis and tryptophan catabolism might influence mortality in septic shock.

In light of these considerations, the aim of this study is to integrate our previous metabolomics results with the information derived from the proteomics analyses in order to have a more complete picture of the changes occurring during one week of treatment at the molecular level and to gain deeper insights into septic shock progression and individual patient’s response. Furthermore, as there are no established approaches or guidelines for integrating metabolomics and proteomics data, we adopted different techniques (i.e. regression analysis methods that performs both variable selection and regularization and partial least squares discriminant analysis) so to compare the features selected and their importance in different models.

Our findings confirmed that non-survivors have different trend in plasma levels of lipid species in comparison to survivors, in line with our previous work^6^. The novelty of this study is the integration with proteomics data, which enables to highlight key features associated with the outcome. More precisely, our approach for integrating metabolites and proteins showed that the proteins decisive in stratifying the patients are those related to the inflammatory response and the coagulation cascade, which are known to play an important role in septic shock progression, thus reinforcing the feasibility and robustness of our integrative approach.

## Results

### Clinical characteristics of the study population

Patients with severe septic shock enrolled in the multicenter ALBIOS clinical trial^5^, and fulfilling the inclusion/exclusion criteria as previously reported, were analyzed. The baseline characteristics of these 17 patients, including site and cause of infection were reported in Table 1. In 9 patients, the cause of infection was identified at site culture, including gram-negative (4 patients), gram-positive (2 patients) and both gram-negative and gram-positive bacterial infection (gram mix, 2 patients), as well as other microorganisms (mixed, 1 patient). Two patients (one S and one NS), had multiple site of infection (S abdomen and other, NS lungs and other). Patients received antibiotic therapy empirically decided during the first 24 hours. Patients were randomized to receive either 20% albumin and crystalloid solutions (10 patients) or crystalloid solutions alone (7 patients) for volume replacement. There was not a significant association between mortality and the kind of fluid administered. On day 28, mortality rate was 47% (8 patients died). Clinical and laboratory variables on day 1 (D1) and day 7 (D7) were reported in Table 2. All the patients were treated according to the standard guidelines internationally accepted for patients with severe sepsis or septic shock at the time of the study^7^.

**Table 1:** Site and cause of the primary infection in survivors (S) and non-survivors (NS). For 8 patients (3 S and 5 NS) the bacterial culture were negative (Negative).

	ALL PATIENTS	S	NS
Age (years)	66.1 ± 13.9	63. 8 ± 16.6	67.9 ± 12.5
BMI (kg/m^2^)	27 ± 3.9	27.5 ± 3.9	27.9 ± 3.2
**Source of infection**			
Lungs [no. (%)]	6 (35%)	1 (11%)	5 (63%)
Abdomen [no. (%)]	8 (47%)	4 (44%)	2 (25%)
Genitourinary [no. (%)]	5 (29%)	5 (56%)	0 (0%)
Other [no. (%)]	3 (18%)	1 (11%)	2 (25%)
**Type of infection**			
Negative [no. (%)]	8 (47%)	3 (33%)	5 (63%)
Mixed [no. (%)]	1 (6%)	0 (0%)	1 (13%)
Gram positive [no. (%)]	2 (12%)	1 (11%)	1 (13%)
Gram negative [no. (%)]	4 (24%)	3 (33%)	1 (13%)
Gram mix [no. (%)]	2 (12%)	2 (22%)	0 (0%)

No statistically significant differences were found between the two groups.

**Table 2:** Clinical and laboratory variables at D1 and D7 for the 17 patients, divided in survivors (S, 9 pts) and non-survivors (NS, 8 pts).

	D1		D7	
	S	NS	S	NS
Heart Rate (bpm)	103.5 ± 28.4	106.1 ± 12.8	80.4 ± 11.0	91.1 ± 8.7
Mean Arterial Pressure (mmHg)	76. 6 ± 18.3	72.0 ± 11.0	96.3 ± 13.8*	78.4 ± 12.0*
Central Venous Pressure (mmHg)	11.4 ± 5.8	11.5 ± 4.4	7.9 ± 5.1	8.8 ± 2.1
Urine output (mL/day)	2556.1 ± 918.4	1840.0 ± 1652.8	3705.6 ± 1580.2*	1737.5 ± 1478.9*
FiO_2_ (%)	59.7 ± 12.4	56.3 ± 20.8	40.6 ± 8.5	46.3 ± 23.4
ScvO_2_ (%)	73.3 ± 11.6	78.5 ± 7.5	77.1 ± 7.3	77.9 ± 4.5
PvCO_2_ (mmHg)	46.8 ± 5.7	47.8 ± 4.9	50.6 ± 5.2	46.5 ± 7.8
PaCO_2_ (mmHg)	42.3 ± 6.3	44.3 ± 6.4	45.3 ± 3.9	41.6 ± 8.3
PvO_2_ (mmHg)	43.3 ± 4.6	46.5 ± 7.4	44.1 ± 5.9	44.6 ± 5.9
PaO_2_ (mmHg)	122.2 ± 61.0	98.5 ± 32.0	126.9 ± 29.8	115.1 ± 61.0
PEEP (cmH_2_O)	8.5 ± 2.5	5.3 ± 5.4	8.9 ± 2.4	8.6 ± 3.4
Lactate (mmol/L)	3.0 ± 1.6	5.0 ± 2.3	1.4 ± 0.5	2.4 ± 2.2
Platelets (×10^3^/mm^3^)	63.9 ± 35.4	61.4 ± 68.1	112.0 ± 67.2	80.3 ± 50.9
Creatinine (mg/dL)	2.7 ± 0.9	2.1 ± 1.3	1.8 ± 1.7	1.8 ± 1.5
Biluribin (mg/dL)	1.7 ± 0.9	5.0 ± 4.8	1.9 ± 1.3	9.1 ± 10.8
Presepsin (µg/L)	1486 ± 1256	2673 ± 2351	830 ± 458	4969 ± 5826
Renal Repl Therapy (RRT)[no. (%)]	0 (0%)	2 (25%)	1 (11%)	3 (38%)
Ventilatory Support [no. (%)]	9 (100%)	8 (100%)	4 (44%)	7 (88%)
**Sofa score**				
Overall	11.3 ± 2.4	12.4 ± 3.2	5.0 ± 2.1	9.3 ± 5.1
Respiratory System	2.4 ± 1.0	2.4 ± 1.3	1.2 ± 0.8	1.9 ± 1.0
Coagulation	2.3 ± 0.9	2.5 ± 1.4	1.6 ± 1.1	1.9 ± 1.2
Liver	1.1 ± 0.9	1.9 ± 1.2	1.0 ± 1.0	2 ± 1.8
Cardiovascular System	3.6 ± 0.5	3.6 ± 0.5	0.0 ± 0.0	1.1 ± 1.5
Renal System	1.9 ± 0.6	2.0 ± 1.7	1.2 ± 1.5	2.4 ± 1.8

Data are presented as mean ± SD or as frequency. Mean Arterial Pressure and Urine output (marked with *) at D7 were significantly different between the two groups (p-value < 0.05 Wilcoxon rank-sum test).

### Changes in protein abundances between groups

A multi-iTRAQ experiment was designed to compare the plasma protein profile between S and NS patients. Criteria for proteins selection are described in details in the Supplemental Methods. In total, from an average of 3000 proteins detected, 132 were selected for further analyses, i.e. only proteins detected with at least two unique peptides in all the 6 iTRAQ experiments. According to Gene Ontology analysis (Slim Biological Process analysis, p < 0.05), these proteins were classified into blood coagulation, complement activation, vitamin transport, cell-cell adhesion, proteolysis and nucleobase-containing compound metabolic processes. For the significant proteins, extended name and main functions are reported in Table S1.

**Figure 1:**
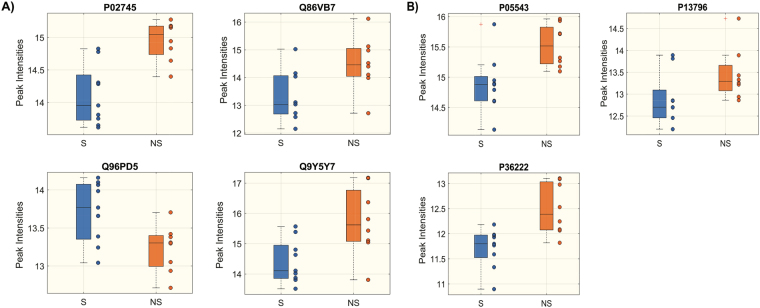
Boxplot of protein peak intensities significantly different between S (blue) and NS (orange) at D1 (**A**) and D7 (**B**) (Wilcoxon rank-sum test p < 0.05, FDR <0.15). Distribution of differences is shown as box-plot. Each plot represents a different protein: P02745, Complement C1-q subcomponent subunit A; Q86VB7, Scavenger receptor cysteine-rich type 1 protein M130; Q96PD5, N-acetylmuramoyl-L-alanine amidase; Q9Y5Y7, Lymphatic vessel endothelial hyaluronic acid receptor 1; P05543, Thyroxine-binding globulin; P13796, Recombinase Flp protein; P36222, Chitinase-3-like protein 1.

We first assessed by univariate analysis if protein levels were significantly different between S and NS separately at the two time points (Wilcoxon rank-sum test p < 0.05, FDR < 0.15). Proteins P02745, Q86VB7, Q96PD5 and Q9Y5Y7 were significantly different between S and NS at D1 and proteins P05543, P13796 and P36222 at D7 (Fig. 1, extended names of the proteins are reported in the legend). Proteins values were reported as normalized peak intensities. As illustrated in the Supplemental material, the raw peak intensities were log2 transformed and LOESS normalized against mean global intensity from all 6 iTRAQ™ 8-plex experiments.

### Time trend variation of proteins and metabolites

**Figure 2:**
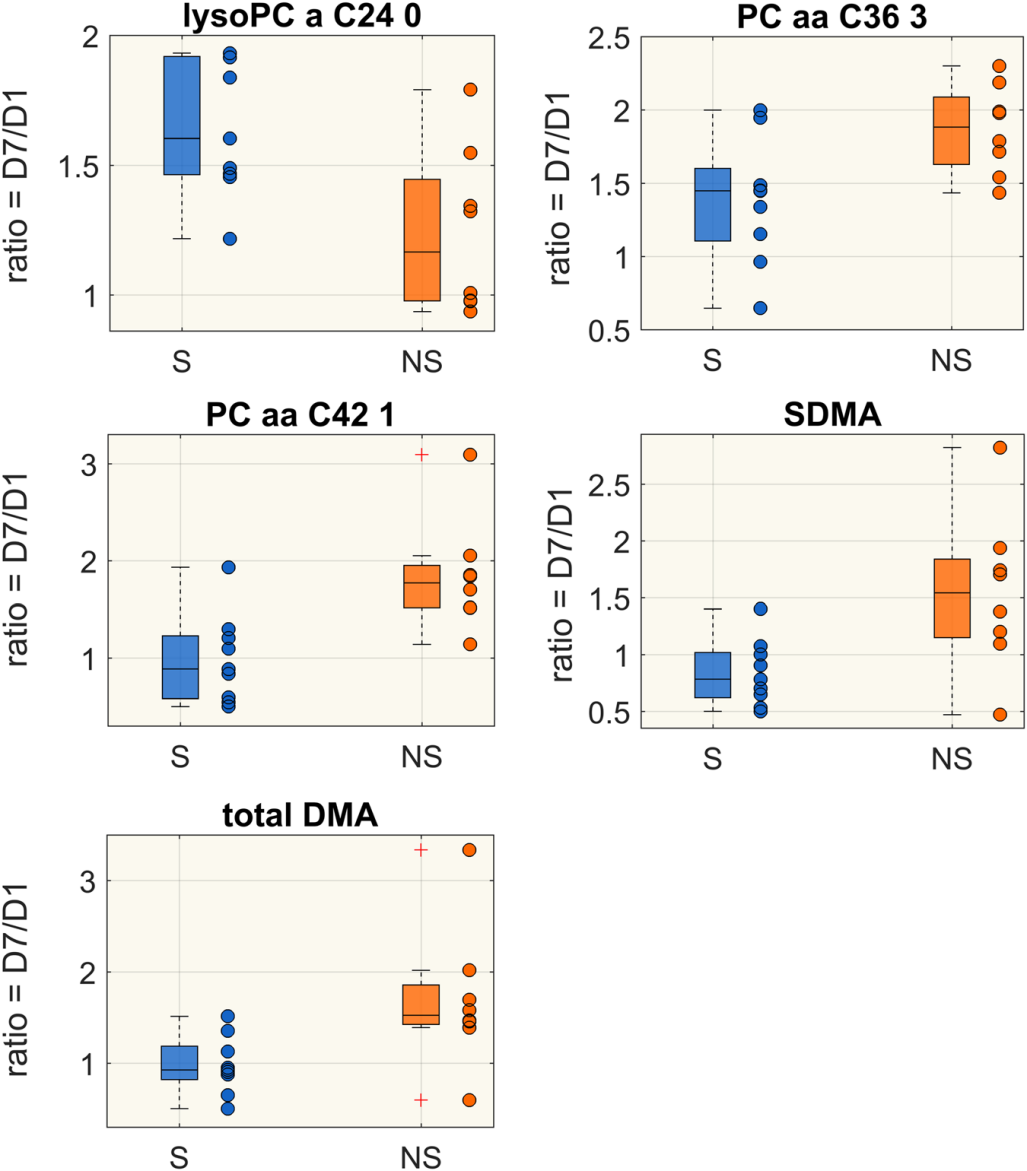
Boxplot of the ratio D7/D1 of metabolite concentrations (µM) significantly different between S (blue) and NS (orange) (Wilcoxon rank-sum test p < 0.05, FDR <0.15).

**Figure 3:**
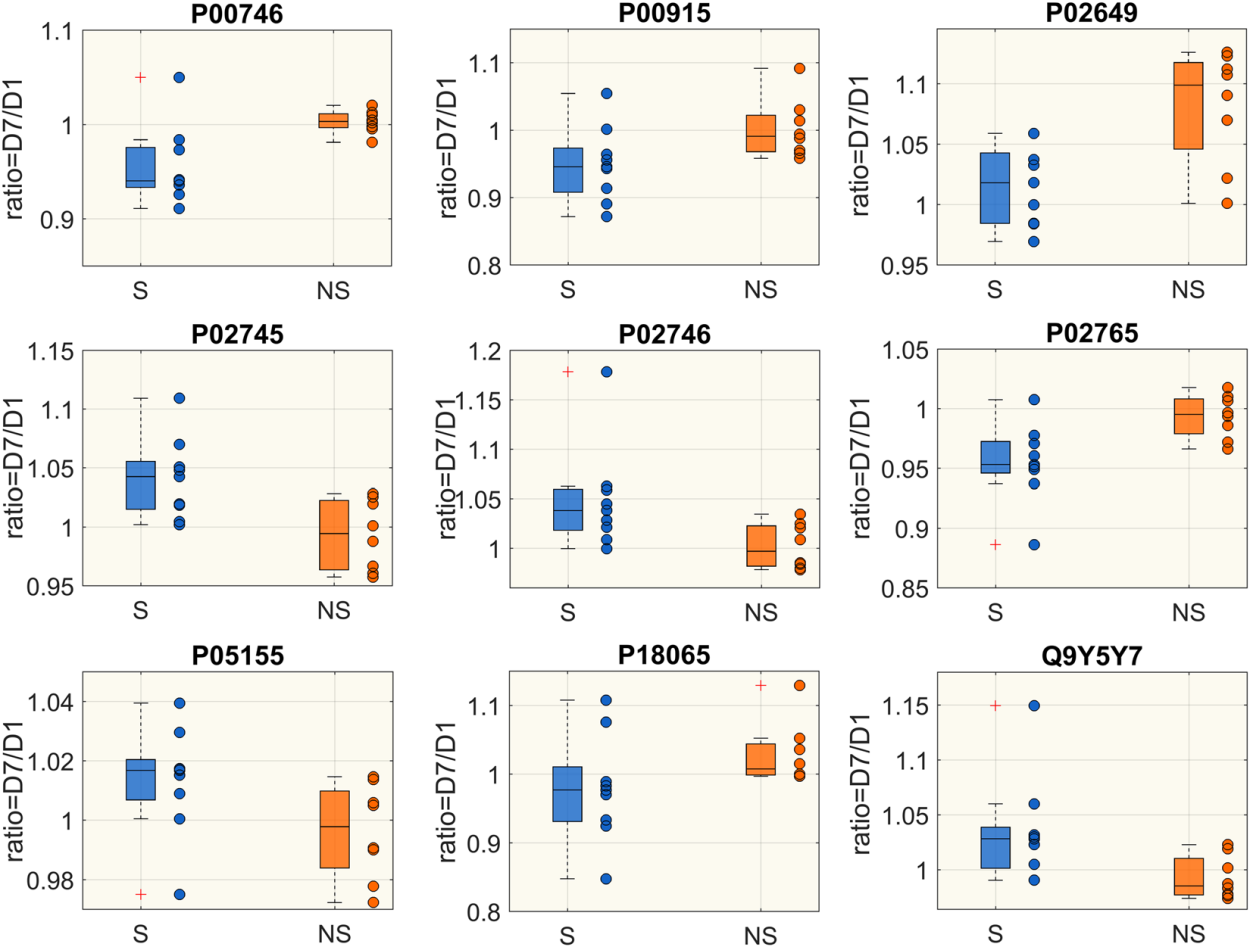
Boxplot of the ratio D7/D1 of protein normalized peak intensities, which are significantly different between S (blue) and NS (orange) (Wilcoxon rank-sum test p < 0.05, FDR <0.15). Each plot represents a different protein: P00746, Complement factor D; P00915, Carbonic anhydrase 1; P02649, Apolipoprotein E; P02745, Complement C1q subcomponent subunit A; P02746, Complement C1q subcomponent subunit B; P02765, Alpha-2-HS-glycoprotein; P05155, Plasma protease C1 inhibitor; P18065, Insulin-like growth factor-binding protein 2; Q9Y5Y7, Lymphatic vessel endothelial hyaluronic acid receptor 1.

Changes in proteins abundance from D1 to D7 within the same group were also assessed; 14 proteins significantly changed from D1 to D7 in the NS group and 10 in the S group (Mann-Whitney t-test p < 0.05, FDR < 0.15). Among them, 9 were significantly different from D1 to D7 in both groups. Temporal trends in the two groups were reported in Table 3. Proteins abundances were expressed as normalized peak intensities. Differences in the ratio D7/D1 between S and NS patients for metabolites and proteins are shown in Figs 2 and 3. Five metabolites, belonging to lipid species and biogenic amines class, and 8 proteins were significantly different between the two groups.

**Table 3:** Values of protein abundance in survivors (S) and non-survivors (NS) at D1 and at D7.

S					NS			
	D1	D7		TREND	D1	D7		TREND
P00751	16.704 (16.203,17.223)	16.308 (16.183,16.619)		↓	17.004 (16.665,17.101)	16.630 (16.122,16.759)	*	↓
P01011	17.046 (16.880,17.197)	16.799 (16.493,17.059)		↓	17.292 (16.897,17.452)	16.509 (16.228,16.994)	*	↓
P02649	15.413 (14.987,15.639)	15.691 (15.234,16.187)		↑	15.222 (15.077,15.757)	16.347 (16.001,16.892)	*	↑
P02741	17.228 (16.847,18.673)	15.921 (15.482,16.165)	*	↓	18.037 (17.845,18.162)	16.385 (15.815,16.683)	*	↓
P02750	17.099 (16.557,17.530)	16.510 (15.985,16.653)	*	↓	17.445 (16.468,17.906)	16.611 (15.951,17.189)	*	↓
P06681	15.394 (15.059,15.611)	14.951 (14.627,15.195)	*	↓	15.579 (15.479,15.909)	15.446 (15.129,15.582)	*	↓
P07358	15.294 (14.675,15.771)	14.908 (14.361,15.434)		↓	14.573 (14.521,15.862)	14.434(14.298,15.415)	*	↓
P07360	15.731 (15.366,16.235)	15.434 (15.343,15.690)	*	↓	15.889 (15.530,16.313)	15.490 (15.107,15.969)	*	↓
P15169	14.701 (14.506,15.917)	14.482 (14.119,15.433)		↓	14.922 (14.082,15.805)	14.641 (13.806,15.523)	*	↓
P18428	15.553 (14.594,15.776)	14.077 (13.318,14.515)	*	↓	15.485 (14.620,15.728)	14.378 (13.827,14.895)	*	↓
P22792	14.339 (14.097,14.900)	14.112 (13.789,14.686)	*	↓	14.521 (14.228,14.798)	14.190 (13.916,14.551)	*	↓
P25311	16.106 (15.665,16.971)	17.427 (16.729,17.668)	*	↑	16.466 (15.381,17.172)	17.398 (16.155,17.793)	*	↑
P36222	13.330 (12.748,14.194)	11.798 (11.524,11.972)	*	↓	14.467 (14.065,14.723)	12.388 (12.076,13.035)	*	↓
P49908	13.549 (13.239,14.047)	14.240 (14.188,14.828)	*	↑	13.163 (12.914,13.674)	13.938 (13.582,14.338)	*	↑
Q15582	14.452 (13.352,14.611)	13.777 (12.819,14.227)	*	↓	14.157 (13,277, 14,384)	14.079 (13.147, 14.143)		↓

Significant differences between D1 and D7 are marked with * (Wilcoxon sign-rank test p < 0.05, FDR < 0.15). Plasma concentrations are expressed as peak intensities and shown as median (25, 75 percentiles). Acronyms: P00751, Complement factor B; P01011, Alpha-1-antichymotrypsin; P02649, Apolipoprotein E; P02741, C-reactive protein; P02750, Leucine-rich alpha-2-glycoprotein; P06681,Complement C2; P07358, Complement component C8 beta chain; P07360, Complement component C8 gamma chain; P15169, Carboxypeptidase N catalytic chain; P18428, Lipopolysaccharide-binding protein; P22792, Carboxypeptidase N subunit 2; P25311, Zinc-alpha-2-glycoprotein; P36222, Chitinase-3-like protein 1; P49908, Selenoprotein P; Q15582, Transforming growth factor-beta-induced protein ig-h3.

### Multivariate analysis

#### Regression model from targeted metabolomics data

We used regression models with the aim of identifying the set of features which were mostly associated to the target class, i.e. the non-survivors (NS). The coefficients of the models obtained from metabolomics concentrations only are reported in Table S5.

**Figure 4:**
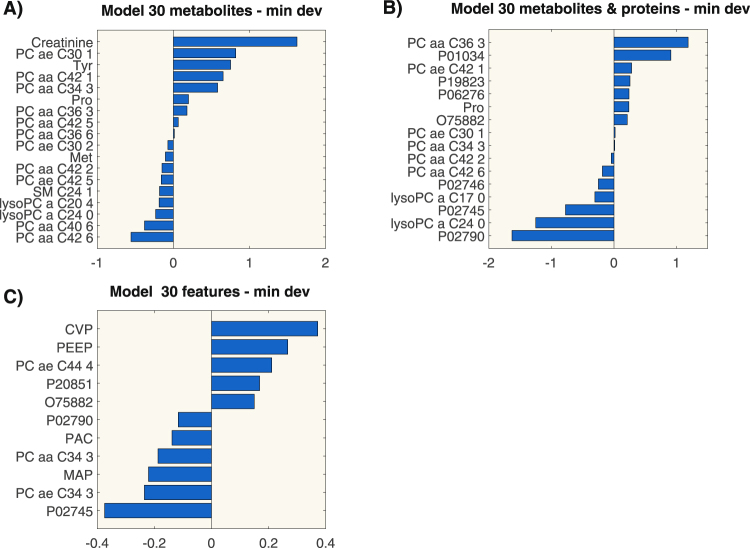
Coefficient values of the logistic regression models built according to the criterion of minimal deviance on the first 30 ranked features. Each panel represents a regression model built on metabolomics data only (panel A), metabolomics and proteomics data (panel B) and on omics data and clinical parameters (panel C). Acronyms: P01034, Cystatin-C; P19823, Inter-alpha-trypsin inhibitor heavy chain H2; P06276, Cholinesterase; O75822, Attractin; P02746, Complement C1q subcomponent subunit B; P02745, Complement C1q subcomponent subunit A; P02790, Hemopexin; P20851, C4b-binding protein beta chain; CVP: Central Venous Pressure; PEEP: Positive End-expiratory pressure; PAC: PaCO_2_; MAP: Mean Arterial Pressure.

Three metabolites were selected in all models: two diacyl-phosphatidylcholine species (PC aa C42:6, PC aa C36:6) and tyrosine. Figure 4A shows the coefficient values of the model built according to the criterion of minimal deviance on the first 30 ranked features. All the obtained models correctly classified the observations in the testing set.

### Regression model from targeted metabolomics and proteomics data

We built the regression models combining metabolomics and proteomics data, as described in the methods section. The coefficients of the models were reported in Table S6. Figure 4B shows the coefficient values of the model built according to the criterion of minimal deviance on the first 30 ranked features. From Tables 4 and S6 we can notice that lysophosphatidylchiline C24:0 (lysoPC a C24:0) and the complement C1q A chain (protein P02745) were selected by all models. Moreover, two diacyl-phospatidylcholine species (PC aa C36:3 and PC aa C42:6) were again selected in these models, and their coefficients maintain the same sign as in previous ones. All the obtained models correctly classified the observations in the testing set.

**Table 4:** Values of VIP scores of the PLS-DA models built on the first 10 and 20 ranked features and the coefficient values of LDA models.

METABOLITES	VIP PLS-DA 20	PLS-DA 10	LDA	METABOLITES & PROTEINS	VIP PLS-DA 20	PLS-DA 10	LDA	METABOLITES PROTEINS CLINICAL DATA	VIP PLS-DA 20	VIP PLS-DA 10	LDA
PC aa C42:1	1.633	—	—	P02745	1.438	1.576	−4.822	P02745	1.681	1.620	−15.017
PC aa C36:3	1.400	1.516	10.290	PC aa C36:3	1.367	—	—	P02790	1.455	1.412	1.349
lysoPC a C24:0	1.372	1.411	0.200	lysoPC a C24:0	1.324	1.433	−2.041	PC ae C44 4	1.334	—	—
lysoPC a C17:0	1.233	—	—	P19823	1.282	—	—	PvCo_2_	1.308	—	—
PC aa C42:6	1.159	1.120	1.785	PC aa C42:6	1.267	—	—	PEEP	1.235	1.053	5.906
PC ae C30:1	1.137	—	—	P02746	1.249	—	—	PaCO_2_	1.229	—	—
PC aa C34:3	1.118	—	—	P02790	1.223	—	—	PC aa C34 3	1.124	1.030	0.172
Tyr	1.096	1.042	7.865	P05543	1.053	1.092	1.627	FiO_2_	1.061	—	—
Pro	1.058	—	—	PC aa C34:3	1.035	—	—	Creatinine	0.986	—	—
Creatinine	0.965	—	—	PC aa C42:2	0.936	1.006	0.729	Urine Output	0.984	0.794	8.590
PC ae C42:1	0.915	0.827	−3.442	PC ae C42:1	0.909	—	—	P05543	0.896	—	—
PC ae C30:2	0.858	0.836	6.010	SM OH C16:1	0.876	0.782	−0.979	MAP	0.847	0.825	−1.140
PC aa C42:2	0.815	0.886	−6.153	O75882	0.866	0.920	0.961	Bilirubine	0.814	0.601	6.668
SM C24:1	0.756	—	—	P22792	0.801	0.826	−2.583	P06276	0.659	—	—
PC aa C42:5	0.753	—	—	P16070	0.745	0.653	1.940	Lactate	0.601	0.268	−1.285
SM OH C16:1	0.639	0.747	−13.680	P20851	0.721	0.438	−0.326	CVP	0.559	—	—
PC ae C34:3	0.600	0.417	—	P06276	0.629	0.706	0.692	Heart Rate	0.552	0.789	6.036
PC aa C36:6	0.585	—	−3.395	Q14520	0.533	—	—	ScvO_2_	0.522	—	—
PC aa C34:4	0.521	0.686	1.921	PC ae C34:3	0.397	—	—	P16070	0.515	0.919	6.023
PC ae C44:4	0.266	—	—	PC ae C44:4	0.232	—	—	pHa	0.266	—	—

The tables summarize the results of the models on metabolites only, metabolites and proteins and the integration of metabolomics, proteomics and clinical data, respectively.

### Classification model from targeted metabolomics, proteomics and clinical data

Finally, we built a model combining metabolomics, proteomics and clinical data as described in the Methods section. The coefficients of the models are reported in Table S7 and Fig. 4C shows the coefficient values of the model built according to the criterion of minimal deviance on the first 30 ranked features. We could notice that also in these models the protein P02745 appeared among the most important predictors, as also confirmed by discriminant analysis (Table 4). Another protein, i.e. Hemopexin (P02790), and the metabolite PC aa C34:3 were also selected by all models. All the obtained models correctly classified the observations in the testing set.

### Discriminant analysis

Table 4 reports the coefficient values of the LDA models and the VIP scores of the PLS-DA models built on the first 10 and 20 ranked features as described in the Methods section.

**Figure 5:**
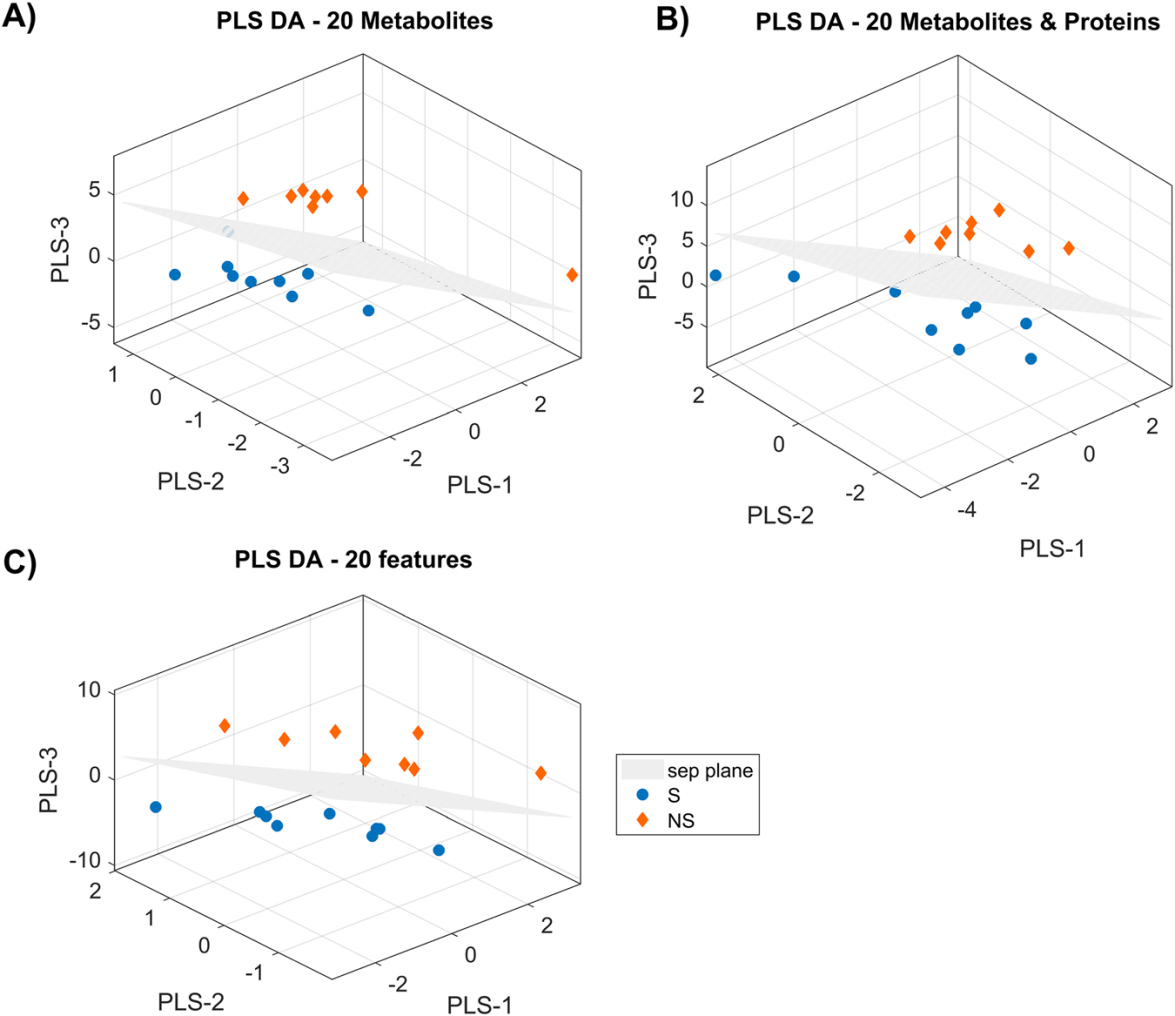
Three-dimensional PLS-DA score plots on the first ranked 20 features for three different models. Each panel represents a model built on metabolomics data only (panel A), metabolomics and proteomics data (panel B) and on omics data and clinical parameters (panel C).

In the metabolites model, PC aa C36:3 occupied the second and first position in the VIP ranking, when considering 20 and 10 features respectively. As for the integrated model, P02745 was in the first position and lysoPC a C24:0 the third (20 feature model) and second (10 feature model), thus confirming the importance of these features already emerged from the regression analysis. In the classification models for omics and clinical data and for metabolomics and proteomics data, we could notice that P02745 occupied the first position followed by another protein, i.e. P02790, in agreement to what emerged from the regression analysis. Three-dimensional PLS-DA score plots on 20 features for the three models are shown in Fig. 5. In all cases, the groups separated perfectly.

## Discussion

The analyses here reported represent a proof-of-concept study, which demonstrates the feasibility of integrating different levels of biological measurements (metabolomics and proteomics) with clinical variables in a longitudinal study providing incremental discriminative capability for mortality risk assessment.

The novelty and the challenge of our approach was to use a large number of variables in a small but well-characterized population and to apply the most suitable data-mining tools to find the best subdivision in the variable domain to separate non-survivors from survivors. The aim was to characterize non-survivors patients according to the variation of biological features that occurred from acute phase (D1) to steady state (D7). Our methodology was based on ranking the species which changed from D1 to D7 in order to find the ones most related to the outcome or those that, although not significant alone, could highlight important association to particular pathways.

In this work, our previous results^6^ and literature findings^8,9^ were confirmed: lipidome alteration was a prominent component of the metabolic phenotype in non-survivors patients with septic shock. A clear bio-signature characterizing non-survivors was their reduction over time in circulating PC species, containing long chain polyunsaturated fatty acid, such as PC aa C42:6, PC aa C40:6, and lysoPC species (Fig. 4A). As already mentioned in our previous work^6^, such lowered PC and lysoPC species could hamper their protective effects^10,11^ and conceivably their reduction, especially lysoPC species, may also promote an excessive immune response^12^. We also observed an imbalance in plasmalogens (PC ae) levels over time between NS and S. Plasmalogens serve as endogenous antioxidants, mediators of membrane structure and dynamics, storage for polyunsaturated fatty acids and lipid mediators^13^. Raising PC ae levels protects human endothelial cells during hypoxia^14^ while their reduction might reflect an increased oxidative imbalance probably due to an excessive systemic inflammatory response^15^.

An exaggerated systemic inflammatory response in non-survivors would be in accordance with the observed overall increase level of methylarginines (total dimethylarigine, DMA; symmetric dimegthylargine, SDMA; Fig. 2) reported to impair macrophage functions^16^ and to be associated with increased mortality in patients with severe sepsis^17^.

Such considerations are in line with proteomics findings. Significant changes in abundance between NS and S mainly involved those proteins which belong to the coagulation, innate immunity and inflammatory response pathways, whose importance has already been recognized in sepsis and septic shock settings^18^. Many works in literature have described modulation in plasma proteins in the complement, coagulation, and fibrinolytic cascades in sepsis survivors and non-survivors^19,20^.

The interplay between these pathways were further highlighted by our integration analyses where lower over time variation of Complement C1-q proteins (P02745, P02746) and Hemopexin (P02790) were the most relevant features for non-survivors. The classical pathway of complement activation represents a link between innate and adaptive immunity. It is activated when Complement C1-q binds a target cell (apoptotic cell or bacteria) expressing membrane PC or antigen/antibody complexes. The activated complement cascade produces inflammatory mediators that stimulate leukocytes and lymphocytes migration. This set of events lead to the lysis of the target cell^21,22^. It is likely that lower variation of C1 complex proteins over one week may indicate worse pathogen clearance and inflammation control in non-survivors compared to survivors. The integration of omics data with clinical variables reinforced the results that low variation in complement C1 and PC metabolic species are associated with poor outcome.

Similarly, lowered Hemopexin (P02790) could imply difficulties in non-survivors to counteract inflammation. Hemopexin is an heme-binding plasma glycoprotein with anti-inflammatory action probably trough the inhibition of inflammatory cytokine production^23,24^. Its decrease over time in non-survivors is in accordance with the low hemopexin serum levels related with poor prognosis of septic shock patients^25^.

Non-survivors were also characterized by an increase in the abundance over-time of Cystatin-C (P01034), whose accumulation in plasma has been related to renal dysfunction^26^. A worsening of renal function estimated by enhanced Cystatin-C rather than by creatinine has been recently described in septic patients with acute kidney injury (AKI) presenting higher Cystatin C levels than non-AKI septic patients^27^. Despite we did not observe significant differences in SOFA score for renal function between the two groups, this value increased from D1 to D7 in non-survivors whereas it decreased in survivors (Table 2)

Integrated models with clinical data are of difficult interpretation, probably due to the fact that we considered a long time interval (7 days). In spite of this, the negative association of MAP (Mean Arterial Pressure) with the outcome was expected since the duration of hypotension or a limited recovery of MAP values is an important predictor of mortality^28,29^. Increase of CVP (central venous pressure) and PEEP (Positive End-expiratory pressure) over time were also indicative of poor outcome in our integrated model. In fact, in the interaction between arterial circulation, organ perfusion and venous circulation, CVP represents the downstream pressure and the increase in downstream pressure may generate congestion^30^.

Age was not considered in our model as our cohort has a limited range in age. Indeed, no significant age differences were found between survivor and non survivors. The range of age in people affected by sepsis is very wide^31^. Consequently, age could be a factor if the cohort under study is very heterogeneous in terms of age and this was not our case.

Importantly, no significant associations between type of fluid administered and mortality was observed in our study population, which is a subset of a larger cohort in the well-defined clinical trial ALBIOS. Indeed, the study on ALBIOS database^5^ showed that in patients with severe sepsis, albumin replacement in addition to crystalloids, as compared with crystalloids alone, did not improve the rate of survival at 28 and 90 days. Thus our results are in line with what observed for the entire database.

We are aware of the limitations of our study. First of all, the size of the datasets used to build the classification models is small. However, we tried to reduce the confounding factors by focusing on a homogeneous group of patients, i.e. severe septic shock ones. We thus hypothesized that the changes observed are mainly related to shock progression and different prognoses. Moreover, we measured metabolites and proteins at two time points only within one week from the diagnosis of septic shock, and biological features with temporal changes out of this time window might provide a more precise insight for the clinical progression of the disease.

Finally, we are aware that selected features before the multivariate analyses certainly affect the successive analyses, but we think that our methodological pipeline may represent an acceptable trade-off approach. The filter approach was necessary to avoid unreliable results, as the features are highly correlated and the logistic regression applied to such type of dataset may not be consistent. Feature reduction is extremely useful when a model is affected by multicollinearity, as in our case^32,33^. In fact, if we have high collinearity and a condition where number of feature *p* is much higher than the number *n* of observations, the algorithm for the coefficients estimate can fail, the overall significance of the model is compromised and the estimate of the regression coefficient can be inaccurate. Therefore, our decisions were driven by the characteristics of our data (i.e. strongly correlated features) and by the size of our dataset: low number of observations (patients) with respect to high number of variables (metabolites, proteins, clinical features)

We must also recall that we are not interested in prediction but in the development of an approach to describe the current dataset and to identify the main pathways involved in pathology progression within the studied cohort.

## Conclusions

In conclusion, for the first time in patients with sepsis, our integrative approach was able to capture possible evolution and variations of metabolic and proteomic signatures during a well-established pathophysiologic manifestation of severe septic shock.

Changes in the abundance of metabolites and proteins within seven days from diagnosis could distinguish survivors and non-survivors among severe septic shock patients. Our data reinforced the emerging evidence that lipidome alterations might influence mortality in septic shock, probably by a cross-talk with inflammatory responses. This study also showed that the combination of proteomics and metabolomics data provides a more complete view of mortality orchestrators, interconnecting complement system and inflammation. Although further validations are needed in a bigger cohort, our results may constitute an important step toward the investigation of combined therapeutic strategies targeted at alteration of both inflammation susceptibility and coagulation cascade.

## Material and Methods

### Study design, patients and clinical data

This pilot retrospective investigation was an ancillary study of the multicenter, randomized Albumin Italian Outcome Sepsis (ALBIOS) clinical trial. It enrolled patients with severe sepsis or septic shock from 100 ICUs in Italy (NCT00707122), as fully described in the original article^34^.

The study was compliant with the 1975 Declaration of Helsinki as revised in 2008, and approved first by the Institutional Review Board of the Fondazione IRCCS Ca’ Granda - Ospedale Maggiore Policlinico, Milan, Italy (coordinating center), and subsequently by the appropriate institutional review boards of all the other participating centers. Written informed consent or deferred consent was obtained from each patient. Patients were managed by the clinical care team according to international guidelines. Patients were randomly assigned to receive either 20% albumin and crystalloid solution or crystalloid solution alone. During the early phase of volume resuscitation, fluids were administered in both groups according to early goal-directed therapy.

Inclusion criteria for the present study are the same as in^6^. We briefly recall them herein: presence of septic shock, serum concentrations of lactate >4 mmol/L, a total SOFA score >8, and availability of plasma samples at day 1 and day 7 in the ALBIOS biobank. In addition, we consider only patients remaining in ICU until 7 up to 14 days from shock onset (until either ICU discharge or death). Exclusion criteria were: presence of active hematological malignancy or cancer, immunodepression, HIV infection, chronic renal failure, or advanced cirrhosis. Such inclusion and exclusion criteria were in accordance with those of the multicenter clinical study, ShockOmics (NCT02141607), and the current study represents a proof of concept study in view of further patient cohort. Only 20 among the 997 patients enrolled in ALBIOS trial and with plasma samples stored in the biobank fulfilled the inclusion criteria. Three out of 20 available patients have been excluded from the present study due to technical problems in handling the plasma samples for proteomics analysis.

The following demographic, clinical and laboratory variables were considered: (i) demographic and anamnestic information collected at ICU admission, at day 1 and at day 7: age (years), sex, body mass index, source of infection and type of infection; (ii) hemodynamic parameters: heart rate (bpm), mean arterial pressure (mmHg), central venous pressure (mmHg), daily urinary output (ml/die); (iii) ventilation parameters: positive end-expiratory pressure (cmH_2_0), inspiratory oxygen fraction –FiO_2_ (%); (iv) blood gas analysis: central venous O_2_ saturation, venous partial pressure of CO_2_, arterial partial pressure of CO_2_, arterial partial pressure of O_2_, central venous partial pressure of O_2_, arterial and venous pH; (v) laboratory and clinical parameters: serum concentrations of creatinine (mg/dL), bilirubin (mg/dL), lactate (mmol/L), platelet count (x10^3^ cells/mm^3^); (vi) Sequential Organ Failure Assessment Score (SOFA) in order to assess daily organ functions^35^, total SOFA score and the sub-scores relating to the respiratory, coagulation, hepatic, cardiovascular, and renal systems; (vii) use of renal replacement therapy (continuous venous-venous hemofiltration), need for ventilatory support. We considered the mortality at day 28 as primary outcome.

For each patient, plasma samples were collected on day 1 (acute phase, D1) and on day 7 (steady state; D7) after study enrolment.

### Proteomics

A multi-iTRAQ (isobaric Tags for Relative and Absolute Quantitation) experiment for simultaneous determination of both the identity and relative abundance of proteins was designed to compare plasma protein pattern expression between S and NS patients. Details about sample preparation, LC-MS/MS analyses, protein identification and data files availability are reported in Supplemental Information. In particular, the following criteria were used for proteins selection: 1) only proteins identified in all iTRAQ experiments were included; 2) contaminant proteins (i.e. the most abundant proteins that should be previously depleted in the immunodepletion process) were removed; 3) only proteins quantified with at least two unique peptides were included. After this selection, 132 proteins were considered for further analyses.

### Targeted metabolomics

A targeted quantitative approach using a combined direct flow-injection and liquid chromatography (LC) tandem mass spectrometry (MS/MS) assay (AbsoluteIDQ 180 kit, Biocrates, Innsbruck, Austria) was applied for the metabolomics analysis of plasma. Methodological details and data preprocessing have been extensively reported in our previous articles^6,36^ and in the Supplemental Information. Briefly, the method combines derivatization and extraction of analytes with the selective mass-spectrometric detection using multiple reaction monitoring (MRM) pairs. Isotope-labeled internal standards are integrated into the platform for metabolite absolute quantification. This strategy allows simultaneous quantification of 186 metabolites (40 amino acids and biogenic amines, 40 acylcarnitines, 90 glycerophospholipids, 15 sphingomyelins, 1 monosaccharide). A metabolite was excluded from further analysis if its concentration did not meet all the following criteria: (1) fewer than 20% of missing values (non-detectable peak) for each quantified metabolite in each experimental group (2) 50% of all sample concentrations for the metabolite had to be above the limit of detection (LOD). In total, 137 of the 186 metabolites expressed as pg/ml were considered for statistical analysis. The list of all the measurable metabolites and the plasma concentration of the metabolites for each patient are provided in Supplemental Information (Tables S2–S4).

### Multivariate analysis

#### Data from targeted metabolomics analyses

The aim of our model was to classify NS patients, in particular to find the species which are mostly associated with the outcome. We built the model on the ratio D7/D1 of metabolite concentrations. Because of the small sample size (17 patients) and the large number of features (137 metabolites), collinearity represents a crucial issue. The method used to reduce the number of features is the minimal-redundancy-maximal-relevance (mRMR)^37^, a filter algorithm based on the mutual information (freely available at http://home.penglab.com/proj/mRMR/). This algorithm ranks features according to their correlation to the outcome (maximum relevance of the feature) and to which information is not explained by the features already selected (minimum redundancy). We considered the first 10, 20 and 30 ranked metabolites to build three different classification models. Data were first normalized (Z score normalization) and the dataset was divided into a training and test set as two third and one third of the observations, respectively.

We adopted two strategies to further select a smaller subset of features. We performed 50 times an elastic net logistic model using a logit function to fit the training set data (*lasso* and *lassoglm* routines in Matlab® enviroment). We decided to use elastic net, as it was proposed to overcome the limits of lasso in cases like our^38^. Indeed, when the number of observation *n* is much higher than the number of features *p*, the lasso selects at most *n* variables before it saturates, because of the nature of the convex optimization problem. This seems to be a limiting feature for a variable selection method. If there is a group of variables among which the pairwise correlations are very high, then the lasso tends to select only one variable from the group and does not care which one is selected. We decide to use 𝛼 = 0.5, i.e. the weight of lasso (L1) versus ridge (L2) optimization, as a good compromise.

We considered a binary classification (S = 0, NS = 1) and the output of the model is a value between 0 and 1, which represents a sort of probability. We then selected the coefficients of the model with the minimal deviance. We also applied another strategy, instead of the coefficients we select the shrinkage parameter λ, corresponding to the model with the minimal deviance, and we used it to fit the elastic net model on the training set to obtain the coefficients of the logistic regression. In both cases, the models were then evaluated on the testing set and the performance were assessed by the number of correct imputations.

Linear Discriminant Analysis (LDA) and Partial Least Squares Discriminant Analysis (PLS-DA) were also used (toolbox freely available at http://www.libpls.net). More precisely, LDA was performed on the first 10 ranked metabolites and the coefficients for the linear boundary between the first and second classes were retrieved. PLS-DA was performed both on the first 10 and 20 ranked metabolites, considering 3 PLS components. Since the groups are unbalanced, the data matrix was weighted centered in order to avoid having a decision boundary shifted towards the most numerous group. The variable importance in projection (VIP) scores, which represent the weights of each feature in PLS-DA model, and the coefficients of LDA were compared to those of logistic regression. Also in this case, the performance of the classification models was evaluated by considering the number of correct imputations.

#### Integration of targeted metabolomics and proteomics data

We built an integrated model by merging targeted metabolomics and proteomics data. Also for proteomics data we computed the ratio D7/D1 for each of the 132 protein peak intensities. To avoid multicollinearity, the mRMR algorithm was applied and the first 50 ranked proteins were selected. These proteins were then combined with the first 50 ranked metabolites and the mRMR was performed again on these new features subset composed of 50 metabolites and 50 proteins. After Z score normalization, we considered the first 10, 20 and 30 ranked features to build the classification models using the two strategies described in the previous paragraph. LDA and PLS-DA were also performed as previously described.

#### Integration of metabolomics, proteomics and clinical data

Finally, we built a comprehensive model which combines targeted metabolomics, proteomics and clinical data. Only continuous clinical variables were considered and their ratio D7/D1 was computed. Total SOFA score and partial SOFA scores were not included to avoid any redundancy. In fact, they are calculated from clinical parameters which are already included in the model and they are thus likely to be correlated. Finally, a total of 17 clinical variables were included. The 17 clinical variables were added to the first 20 ranked features from the set of metabolites and proteins, obtained as previously described. The mRMR was then performed on this subset of features to further reduce the number of features. After Z score normalization, the first 10, 20 and 30 ranked features were selected to build the classification models. LDA and PLS-DA were also performed on this subset of features composed of metabolites, proteins and clinical parameters.

## Electronic supplementary material

Supplemental Information and Data
