# Supplementary material for: An Innovative Approach for The Integration of Proteomics and Metabolomics Data In Severe Septic Shock Patients Stratified for Mortality
Source: Sci Rep. 2018 Apr 27;8:6681. doi: 10.1038/s41598-018-25035-1 (PMC5923340; doi:10.1038/s41598-018-25035-1)
Supplement: Supplementary file 1 — Supplemental Information and Data [file 41598_2018_25035_MOESM1_ESM.docx]

**Supplemental Information**

**AN INNOVATIVE APPROACH FOR THE INTEGRATION OF PROTEOMICS AND METABOLOMICS DATA IN SEVERE SEPTIC SHOCK PATIENTS STRATIFIED FOR MORTALITY**

Authors:

Alice Cambiaghi^1*^, Ramón Díaz^2*^, Julia Bauzá Martinez^2^; Antonia Odena^2^, Laura Brunelli^3^, Pietro Caironi^4,5^, Serge Masson^3^, Giuseppe Baselli^1^, Giuseppe Ristagno^3^, Luciano Gattinoni^6^, Eliandre de Oliveira^2+§^, Roberta Pastorelli^3+^, Manuela Ferrario^1+§^

Affiliations:

^1^ Politecnico di Milano, Milan, Italy

^2^Proteomics Platform - Parc Científic de Barcelona, Barcelona, Spain.

^3^IRCCS-Istituto di Ricerche Farmacologiche Mario Negri, Milan, Italy

^4^ Anestesia e Rianimazione, Azienda Ospedaliero-Universitaria S. Luigi Gonzaga, Orbassano, Italy

^5^ Dipartimento di Oncologia, Università degli Studi di Torino, Turin, Italy

^6^ Department of Anesthesiology, Emergency and Intensive Care Medicine, University of Göttingen, Germany

**INDEX**

**Supplemental Methods:** proteomics analyses by iTRAQ quantitation.

**Supplemental Methods:** targeted metabolomics. Absolute metabolite profiling and filtering criteria.

**Supplemental Methods**: brief description of algorithms used in Matlab environment.

**Supplemental Table S1 -** List of the proteins identified in univariate and multivariate analyses.

**Supplemental Table S2**. . List of the measurable metabolites using the Biocrates Absolute IDQ p180 kit.

**Supplemental Table S3**. Plasma concentration (µM) of quantified metabolites at day 1 for each patient. Survival status at 28 days and 90 days is also reported (S, survivor; NS, non survivor).

**Supplemental Table S4**. Plasma concentration (µM) of quantified metabolites for each patient at day 7 and survival rate at 28 and 90 days (S, survivor; NS, non survivor).

**Supplemental Table S5 -** Coefficient values of the logistic regression models.

**Supplemental Table S6 -** Coefficient values of the logistic regression models for integration of metabolomics and proteomics.

**Supplemental Table S7-** Coefficient values of the logistic regression models for integration of omics data with clinical parameters

**Methods**

**PROTEOMICS ANALYSES BY ITRAQ QUANTITATION**

**STUDY DESIGN**

A multi-iTRAQ experiment was designed to compare the plasma protein pattern expression between survivor (S) and non-survivor (NS) patients. Two time points were analyzed to compare both study groups: day 1 (acute state, D1) and day 7 after diagnosis of septic shock (steady state, D7). Sample from 17 septic shock patients (9 S and 8 NS) and from 5 healthy donors (M1 to M5) were arranged in six iTRAQ™ 8plex experiment. The 5 healthy donors were used for LC-MS normalization purposes.

**SAMPLE PREPARATION**

**Human Plasma depletion.** Collected human plasma samples (30 µl) were depleted to remove the 14 most abundant plasma protein using a Seppro® IgY 14 LC2 inmunoaffinity column (Sigma Aldrich) as per manufacturer’s indications. After depletion, resulting plasma was concentrated and buffer exchanged to 10 mM Tris-HCl/150 mM NaCl (pH 7.4) using centrifugal filters (Millipore 4.5 ml filters, 10 kDa, 4000 g, 15 o C), and then quantified by Micro BCA™ Protein Assay Kit (Thermo Scientific).

**In solution sample digestion.** 35 µg of depleted protein plasma were brought up to 75 µl with 50 mM triethylammonium bicarbonate (TEAB) and then denatured with 9 µl of Rapigest® detergent (Waters) (0.15% w/v in digestion step). Samples were reduced with tris (2-carboxyethyl) phosphine (5,5 mM, 60 min, 60oC), and alkylated with iodoacetamide (25 mM, RT, 30 minutes in the dark). Proteins were digested with 1.4 µg of trypsin per sample for 4 h (Promega, trypsin sequence grade; 37 ^o^C, pH 8.0) and then re-digested for other additional 16 h (37 ^o^C, pH 8.0) with 7 µg of trypsin per sample. After digestion, the resulting solution was acidified with trifluoroacetic acid TFA (1% final concentration, pH<2) and incubated for 60 min at 37^o^C to hydrolyze Rapigest®. The acidified solution was centrifuged at 14000 rpm, and the supernatant peptide mixture recovered and desalted in a C18 tip (P200 Toptip, PolyLC), as per manufacturer's indications. The peptide solution was dried in a SpeedVac system and kept at -20^o^C until used.

**Peptide labeling.** Each iTRAQ run was composed of samples from three septic shock patients both at D1 and D7 (six samples in total), and of samples from two healthy donors as internal standard to test technical reproducibility. C18 Cleaned-up samples were resuspended in 30 μL 500 mM TEAB, to perform iTRAQ labeling (iTRAQ™ 8plex Multiplex kit) according to the product specifications. Briefly, 70 µl of isopropanol were added to each vial of iTRAQ labeling reagent. The content of these vials was transferred to each sample tube, and sample-iTRAQ mixtures were mixed and incubated at room temperature for 2 h to allow the iTRAQ labeling reaction.

An aliquot of each reaction was cleaned up with a C18 homemade stage tip and analyzed by LC-MS/MS to ensure complete labeling. 100 µl of water were added to reaction mixtures in order to quench the iTRAQ reaction and labeled samples were combined and dried down in a SpeedVac system.

**Sample clean-up and fractionation.** Before LC-MS/MS analysis, two clean-up steps were performed on the labelled mixture. In the first clean-up step, the sample was resuspended in 100 μL 1% formic acid (FA) solution, desalted in a C18 tip (P200 Toptip, PolyLC) and dried in a SpeedVac system. In the second clean up step, dried peptides were resuspended in 100 μL 20 % acetonitrile (ACN)/0.1 % FA (pH 2.7-3), cleaned in a strong cationic exchange tip (P200 toptip, PolySULFOETHYL A PolyLC /0.1% FA) and dried in a SpeedVac system.

The sample was then subjected to high pH fractionation with a high pH reversed phase peptide fractionation kit (Pierce, ref.84868) following the manufacturer’s instructions. Briefly the samples were loaded onto a spin column in 0.1% TFA, washed and buffer exchanged with high pH buffer and then eluted in 9 fractions of increasing acetonitrile (ACN) concentration (f1 = 10% ACN; f2 = 12.5% ACN; f3 = 15% ACN; f4 = 17.5% ACN; f5= 20 % ACN; f6 = 22.5% ACN; f7= 25% ACN; f8 = 50% ACN; f9 = 75% ACN). Flow through and wash fractions were pooled and analyzed as FTwash fraction. The fractions (a total of 10) were dried down in a speed-vacuum centrifuge.

**LC-MS/MS ANALYSES**

The 10 dried-down fractions were analyzed in a nanoAcquity liquid chromatographer (Waters) coupled to a LTQ-Orbitrap Velos (Thermo Scientific) mass spectrometer. Tryptic labelled peptides of each fraction were resuspended in 2% ACN/1% FA solution and an aliquot was injected for their chromatographic separation. Peptides were trapped on a Symmetry C18^TM^ trap column (5 µm, Øi, 180 µm x 20 mm; Waters), and separated using a C18 reverse phase capillary column (75 μm Øi, 25 cm, nano Acquity, 1.7μm BEH column; Waters). The gradient used for the elution of the peptides was 2 to 35 % B in 155 minutes, followed by gradient from 35% to 45% in 20 min (A: 0.1% FA; B: 100% ACN, 0.1% FA), with a 250 nL/min flow rate.

Eluted peptides were subjected to electrospray ionization in an emitter needle (PicoTipTM, New Objective) with an applied voltage of 2000V. Peptide masses (m/z 300-1800) were analyzed in data dependent mode where a full Scan MS in the Orbitrap with a resolution of 30,000 full width half maximum (FWHM) at 400 m/z was obtained. Up to 15 most abundant peptides (minimum intensity of 2000 counts) were selected from each MS scan and then fragmented in the HCD (Higher Energy Collision Dissociation) cell, using nitrogen as collision gas, with 40% normalized collision energy. Following, the MS/MS fragmented peptides were analyzed in the Orbitrap with a resolution of 7,500 FWHM at 400 m/z. The scan time settings were: Full MS 250 ms (1 microscan) and MSn 300 ms (2 microscans). Generated raw data files (raw format) were collected with Thermo Xcalibur (v.2.2).

**DATABASE SEARCH**

Thermo Proteome Discover (v.1.4.1.14) was used to search with SequestHT search engine against the SwissProt Human public database (v. March 2015). For each iTRAQ batch, 10 raw files corresponding to the 10 injections from the MS analyses were used to perform a single search against this database. A database search against both a targeted and a decoy database was made to obtain a false discovery rate (FDR), and thus estimate the number of incorrect peptide-spectrum matches which exceed a given threshold. Additionally, to improve the sensitivity of the database searching, the semi-supervised Percolator algorithm was used in order to enhance the discrimination of correct and incorrect peptide spectrum matches. Percolator assigns a q-value to each spectrum, which is defined as the minimal FDR at which the identification is deemed to be correct. These q-values are estimated using the distribution of scores from a decoy database search. A quantification method for iTRAQ™ 8-plex mass tags optimized for Thermo Scientific Instruments was applied to obtain the reporter ion intensities. The following search parameters were applied:

• Database/Taxonomy: SwissProt Human (v. March2015) plus contaminants

• Enzyme: Trypsin

• Missed cleavage: 2

• Fixed modifications: Carbamidomethyl of cystein, iTRAQ8plex (N-term)

• Variable modifications: oxidation of methionine, iTRAQ8plex (Y), iTRAQ8plex (K)

• Peptide tolerance: 10 ppm and 0.1 Da (respectively for MS and MS/MS spectra)

• Percolator: Target FDR (Strict) 0.01; validation based on: q-value<0.01

**DATA PRE PROCESSING**

Reporter intensities from Proteome Discoverer quantitation files were used to perform iTRAQ quantitation. Within each iTRAQ™ 8plex experiment, reporter ion intensities of each individual peptide were summed from 10 injected fractions (LC-MS run), log2 transformed and then LOESS normalized against mean global intensity from the all 6 iTRAQ™ 8plex experiments. Protein abundance was defined as the mean of the normalized intensities values belonging to the given protein for each reporter. Data validation and normalization was performed by R, v3.1.2 and Inferno RND software v 1.0 (graphical front-end to R for common data analysis; Pacific Northwest National Laboratory; US Department of energy).

**Criteria for proteins selection.** The following procedure was used for proteins selection: 1) only proteins identified in all six iTRAQ experiments were included; 2) contaminant proteins (i.e. the most abundant proteins that should be previously depleted in the immunodepletion process) were removed; 3) only proteins quantified with at least two unique peptides were included. After this selection, 132 proteins were considered for further analyses.

**Quality control.** In order to assess experimental variability among the six iTRAQ experiments, two plasma samples from five healthy controls (M1 to M5) were included in each iTRAQ experiment and were used to test for significant differences or bias. For each of the five control samples (M1-M5), the differences in measured proteins abundance between the pairs of replicates were computed (e.g. for sample labeled as M1 we computed Δ RUN 1-5 = M1 RUN1 – M1 RUN5). For each of the series of differences, the Lillierfors test was performed against the null hypothesis of Gaussian distribution and the Student test against the null hypothesis that the series have mean value equal to zero. In this way, we statistically verify if the differences between runs are randomly distributed around zero. For each of the series, both tests have a p-values < 0.05, thus we cannot reject the hypothesis that differences are normally distributed around zero. This implies that there are no biases.

The whole mass spectrometry proteomics data have been deposited to the ProteomeXchange Consortium (http://proteomecentral.proteomexchange.org/cgi/GetDataset) via the PRIDE partner repository with the data set identifier PXD006730, in addition to the Excel file with the 132 proteins selected.

**STATISTICAL ANALYSES**

Patients were stratified into 9 survivors (S) and 8 non-survivors (NS) according to their mortality 28 days after study enrollment. For each time point (D1 and D7) at which the blood samples were collected, we considered 26 clinical parameters (Table 1), 137 metabolites concentrations (µM) and 132 proteins values (expressed as peak intensities), for a total of 294 features.

For each of the 132 protein abundances, comparisons between S and NS at D1 and D7 were performed using a 2-way ANOVA. The observations were grouped according to two factors: run and outcome (S/NS). Two separate tests were performed, one for D1 and one for D7. Only those proteins associated to p-val_OUTCOME_<0.05 and p-val_OUTCOME*RUN_>0.05 were considered.

On these proteins, we compared the peak intensities measured at D1 and D7 of S and NS groups by means of Wilcoxon rank-sum test. To overcome the problem of the large number of statistical comparisons, we computed also the false discovery rate (FDR), assessed after the bootstrapping procedure. Results were considered statistically significant when p-value <0.05 and FDR <0.15.

Comparisons between D1 and D7 within the same group were performed with a 2-way ANOVA for repeated measures. The repeated measures model included outcome, run, and day (D1 or D7), where DAY represents the repeated factor. Those proteins (3 in total) which are affected by the run were excluded from further analyses. Post hoc comparisons were then performed on the remaining proteins and their trend from D1 to D7 was compared by means of the paired Mann-Whitney t-test. Finally, we compared the ratio D7/D1 for metabolite concentrations and protein peak intensities between S and NS by Wilcoxon rank-sum test. Also in this case, the FDR was computed as previously described and results were considered statistically significant when p-value <0.05 and FDR <0.15.

**EXPRESSION VALIDATION OF PROTEIN P02745 BY ELISA ASSAY**

To confirm the mass spectrometry data and to further strengthen our results, we selected the protein P02745, to verify the reliability of peak intensities estimated by iTRAQ. Protein P02745 resulted significant in most all the models explored. The concentration of Complement C1-q (P02745) was measured by sandwich ELISA assay (ab170246 Kit, Abcam) according to the manufacturer instructions. Briefly, all the 39 plasma samples (17 patients with sepsis on D1 and D7, and 5 healthy subjects) were analyzed in duplicate, as well as the standard curve. 50 µL of each sample and Complement C1-q standard were added to the wells and incubated for 2 hours. Then the wells were washed five times and 50 µL of biotinylated Complement C1-q Detector Antibody were added to each well and incubated for 1 hour. After wells washing, 50 µL of Streptavidin-Peroxidase Conjugate were added and incubated for 30 min. A final washing step was done and 50 µL of Chromogen Substrate was added and incubated for 10 min. 50 µL of Stop Solution were added immediately before reading absorbance at 450nm (ELx800, BIO-TEK). The Complement C1-q concentration in the samples was measured by comparing the O.D. lectures with the ones of the Standard Curve (R = 0.993). The concentration obtained by ELISA are in line with what already seen by mass spectrometry.

**TARGETED METABOLOMICS**

**Absolute Metabolite profiling and filtering criteria**

Targeted metabolomics analysis of plasma samples from study subjects was performed using the Biocrates AbsoluteIDQTM p180 kit (Biocrates Life Science AG, Innskruck, Austria). This validated targeted assay allows for simultaneous detection and quantification of metabolites in biological samples in a high-throughput manner. The metabolite extracts were processed following the instructions by the manufacturer and analyzed on a triple-quadropole mass spectrometer (AB SCIEX triple-quad 5500) operating in the multiple reaction monitoring (MRM-MS) mode. The assay is based on PITC (phenylisothiocyanate)-derivatization in the presence of internal standards for the analysis of aminoacids and biogenic amines resolved and quantified by liquid chromatography- tandem mass spectrometry (LC-MS/MS) using scheduled MRMs. Subsequent flow injection analysis tandem mass spectrometry (FIA-MS/MS) was performed to analyze acylcarnitines, glycerophospholipids, hexose. MRM detection was used for quantification applying spectra parsing algorithm integrated into the MetIQ software (Biocrates Life Science AG, Innskruck, Austria). Concentrations were calculated and evaluated by comparing measured analytes in a defined extracted ion count section to those of specific labeled internal standards or non-labeled ones, provided by the kit. The measurements are made in a 96-well format. Seven calibration standards, five quality control samples, three zero samples (methanol) and one blank (solvents) are integrated into the plate. The limit of detection for the individual metabolites is set three times the value of the “zero samples”. The average coefficient of variation of the metabolites among the biological replicates was 30%. This variation is the sum of biological and technical variation. Based on the five quality controls (QCs) included in the mass spectrometric analysis to monitor the instrumental performances and evaluate the quality of the data, the CV was below 15% (technical variation). For glycerophospholipids, the precise position of the double bonds and the distribution of the carbon atoms in different fatty acid side chains cannot be determined with this technology. Consequently, the detected MRM signal is a sum of several isobaric/isomeric lipds. For example, according to LIPID MAPS database ([www.lipidmaps.org](http://www.lipidmaps.org)) the signal of PCaa C36:6 can arise from at least 15 different lipid species that have different fatty acid composition (e-g. PC 16:1/20:5 versus PC 18:4/18:2, various position of fatty acid sn-1/sn-2 (e.g. PC 18:4/18:2 versus PC 18:2/18:4) and different double bond positions and stereochemistry in those fatty acid chains (e.g. PC(18:4(6Z,)Z,12Z,15Z)/18:2(9Z,12Z) versus PC (18:4(9E,11E,13E,15E)/18:2(9z,12Z)).

Lipid side-chain composition is abbreviated as Cx:y, where x denotes the number of carbons in the side chain and y the number of double bonds. The nature of fatty acids linkage is expressed as aa for diacyl or ae for acyl-alkyl. For example, PCaaC32:1 denotes diacyl-phosphatidylcholine with 32 carbons in the two fatty acids side chains and a single double bond in one of them. The list of all the measurable metabolites and the plasma concentration of the metabolites for each patients are provided in supplemental **table S2, table S3** and **table S4**.

**Algorithms used in Matlab environment.**

lassoglm and fitcdiscr are standard rootine in Matlab, mrmr_mid_d is freely available

% X: matrix of data (N observation x J variables); p: cases of the training set

% class: binary class

for i=1:50 % 50 times

[B,FitInfo] =lassoglm(X(p,:),class(p),'binomial', 'link','logit','CV',2,'Alpha',0.5,'PredictorNames',predictor_names);

pos=FitInfo.Index1SE;

ERR(i,1)=FitInfo.Deviance(pos);

Inter(i,1)=FitInfo.Intercept(pos);

B_all(:,i)=B(:,pos);

predictors{i,1}=predictor_names(find(B(:,pos)));

I_predict{i,1}=find(B(:,pos));

lambda(i,1)=FitInfo.Lambda1SE;

end

%selection of the model with the minimal deviance

[i j]=min(ERR);

Lam=lambda(j);

[B_lam,FitInfo_lam] =lassoglm(X,class,'binomial', 'link','logit','CV',2,'Alpha', 0.5, 'Lambda', Lam, 'PredictorNames', predictor_names);

%variable selected by this elastic net model

p_lambda=find(B_lam);

B1 = [FitInfo_lam.Intercept;B_lam(p_lambda)];

%LDA model

%X: matrix of parameters

class=[zeros(9,1); ones(8,1)]; %S=0; NS=1

MdlLinear = fitcdiscr(X,class);

Coeff = MdlLinear.Coeffs(2,1).Linear; %coefficients

% mRMR algorithm for feature selection

%X: matrix of parameters

X =data;

m=mean(X,1);

sd=std(X,0,1);

th1=m-sd;

th2=m+sd;

X_disc=nan(size(X));

N=length(data)

for i=1:length(th1)

x=X(:,i);

j=find(x<=th1(i));

if ~isempty(j); X_disc(j,i)=-1;end

j=find(x>th1(i)& x<=th2(i));

if ~isempty(j);X_disc(j,i)=0;end

j=find(x>th2(i));

if ~isempty(j); X_disc(j,i)=1;end

end

[pos] = mrmr_mid_d(X_disc, class, N);

mRMR_all_nomi=N(pos);

| **UniProt ID** | **Protein name** | **Main functions** |
| --- | --- | --- |
| **O75882** | Attractin | Inflammatory response |
| **P00746** | Complement factor D | complement activation |
| **P00751** | Complement factor B | complement alternate pathway, innate immunity |
| **P00951** | Carbonic anhydrase 1 | bicarbonate transport |
| **P01011** | Alpha-1-antichymotrypsin | acute phase inflammatory response |
| **P02649** | Apolipoprotein E | lipid metabolism and transport |
| **P02649** | Apolipoprotein E | acute phase |
| **P02745** | Complement C1q subcomponent subunit A | complement pathway, innate immunity |
| **P02746** | Complement C1q subcomponent subunit B | complement pathway, innate immunity |
| **P02750** | Leucine-rich alpha-2-glycoprotein | angiogenesis, endothelial cell proliferation |
| **P02765** | Alpha-2-HS-glycoprotein | acute-phase response |
| **P02790** | Hemopexin | Host-virus interactions |
| **P05155** | Plasma protease C1 inhibitor | blood coagulation |
| **P05543** | Thyroxine-binding globulin | negative regulation of endopeptidase activity, thyroid hormone transport |
| **P06276** | Cholinesterase | contributes to the inactivation of the neurotransmitter acetylcholine |
| **P06681** | Complement C2 | complement pathway, innate immunity and immunity |
| **P06727** | Apolipoprotein A-IV | lipid transport |
| **P07358** | Complement component C8 beta chain | complement pathway, innate immunity and immunity, cytolysis |
| **P07360** | Complement component C8 gamma chain | complement pathway |
| **P01034** | Cystatin-C | cysteine proteinases inhibitor |
| **P13769** | Recombinase Flp protein | DNA recombination and integration |
| **P15169** | Carboxypeptidase N catalytic chain | peptide metabolic process, protein processing |
| **P18065** | Insulin-like growth factor-binding protein 2 | T-cell regulation |
| **P18428** | Lipopolysaccharide-binding protein | lipid transport, innate immunity and immunity |
| **P19823** | Inter-alpha-trypsin inhibitor heavy chain H2 | serine-type endopeptidase inhibitor activity |
| **P20851** | C4b-binding protein beta chain | controls the classical pathway of complement activation |
| **P22792** | Carboxypeptidase N subunit 2 | regulation of catalytic activity |
| **P25311** | Zinc-alpha-2-glycoprotein | stimulation of lipid degradation |
| **P36222** | Chitinase-3-like protein 1 | apoptotic process, inflammatory response |
| **P49908** | Selenoprotein P | response to oxidative stress |
| **Q15582** | Transforming growth factor-beta-induced protein ig-h3 | cell adhesion, sensory transduction |
| **Q86VB7** | Scavenger receptor cysteine-rich type 1 protein M130 | acute-phase response |
| **Q96PD5** | N-acetylmuramoyl-L-alanine amidase | innate immune response, regulation of inflammatory response |
| **Q9Y5Y7** | Lymphatic vessel endothelial hyaluronic acid receptor 1 | receptor activity (transport), metabolic processes |

**Table S1** - List of the proteins identified in univariate and multivariate analyses (alphabetic order) reporting UniProt ID, extended protein names and main functions.

| **mETABOLITE CLASS** | **nUMBER** | **mETABOLITE NAME OR ABBREVIATION** | **Biological relevance**  **(SELECTED EXAMPLES)** |
| --- | --- | --- | --- |
| **Amino acids** | 21 | Alanine, arginine, aspartate, citrulline, glutamine, glutamate, glycine, histidine, isoleucine, leucine, lysine, methionine, ornithine, phenylalanine, proline, serine, threonine, tryptophan, tyrosine, valine | Amino acid metabolism, urea cycle, activity of gluconeogenesis and glycolysis, insulin sensitivity, neurotransmitter metabolism, oxidative stress |
| **Carnitine** | 1 | C0 | Energy metabolism, fatty acid transport and mitochondrial fatty acid oxidation, ketosis, oxidative stress, mitochondrial membrane damage |
| **Acylcarnitine** | 39 | C2, C3, C3:1, C3-OH, C4, C4:1, C4-OH, C5, C5:1, C5:1-DC, C5-DC, C5-M-DC, C5-OH, C6, C6:1, C7-DC, C8, C9, C10, C10:1, C10:2, C12, C12-DC, C14, C14:1, C14:1-OH, C14:2, C14:2-OH, C16, C16:1, C16:1-OH, C16:2, C16:2-OH, C16-OH, C18, C18:1, C18:1-OH, C18:2 |  |
| **Biogenic amines** | 19 | Acetylornithine, asymmetric dimethylarginine, total dimethylarginine, alpha-aminoadipic acid, carnosine, creatinine, histamine, kynurenine, methionine sulfoxide, nitrotyrosine, hydroxyproline, phenylethylamine, putrescine, sarcosine, serotonin, spermidine, spermine, taurine | Neurological disorders, cell proliferation, cell cycle progression, DNA stability, oxidative stress |
| **Lyso-phosphatidylcholines** | 14 | lysoPC a C14:0/C16:0/C16:1/C17:0/C18:0/C18:1/C18:2/C20:3/C20:4/C26:0/C26:1/C28:0/C28:1 | Degradation of phospholipids, membrane damage, signaling cascades, fatty acid profile |
| **Diacyl-phosphatidylcholines** | 38 | PC aa C24:0/C26:0/C28:1/C30:0/C30:2/C32:0/C32:1/C32:2/C32:3/C34:1/C32:2/C34:3/C32:4/C36:0/C36:1/C36:2/C36:3/C36:4/C36:5/C36:6/C38:0/C38:1/C38:3/C38:4/C38:5/C38:6/C40:1/C40:2/C40:3/C40:4/C40:5/C40:6/C42:0/C42:1/C42:2/C42:4/C42:5/C42:6 | Dyslipidemia, membrane composition and damage, fatty acid profile, activity of desaturases |
| **Acyl-alkyl-phosphatidylcholine** | 38 | PC ae C30:0/C30:2/C32:1/C32:2/C34:0/C34:1/C34:2/C34:3/C36:0/C36:1/C36:2/C36:3/C36:4/C36:5/C38:0/C38:1/C38:2/C38:3/C38:4/C38:5/C38:6/C40:1/C40:2/C40:3/C40:4/C40:5/C40:6/C42:0/C42:1/C42:2/C42:3/C42:4/C42:5/C44:3/C44:4/C44:5/C44:6 |  |
| **Sphingomyelins** | 15 | SM (OH) C14:1, SM C16:0, SM C16:1, SM C16:1, SM C18:0, SM C18:1, SM C20:2, SM C22:3, SM (OH) C22:1, SM (OH) C22:2, SM C24:0, SM C24:1, SM (OH) C24:1, SM C26:0, SM C26:1 | Signaling cascades, membrane damage (eg. neurodegeneration) |
| **Hexose** | 1 | H1 | Carbohydrate metabolism |
| **Total** | 186 |  |  |

**Table S2**. List of the measurable metabolites using the Biocrates Absolute IDQ p180 kit.Aa, acyl-acyl; ae, acyl-alkyl; a, lyso; Cx:y, where x is the number of carbons in the fatty acid side chain; y is the number of double bonds in the fatty acid side chain; DC, decarboxyl; M methyl; OH, hydroxyl; PC, phosphatidylcholine; SM, sphingomyelin

**Table S3**. Plasma concentration (µM) of quantified metabolites at day 1 for each patient. Survival status at 28 days and 90 days is also reported (S, survivor; NS, non survivor

| **Metabolite D1** | **#1** | **#2** | **#3** | **#4** | **#5** | **#6** | **#7** | **#8** | **#9** | **#10** | **#11** | **#12** | **#13** | **#14** | **#15** | **#16** | **#17** | **#18** | **#19** | **#20** |
| --- | --- | --- | --- | --- | --- | --- | --- | --- | --- | --- | --- | --- | --- | --- | --- | --- | --- | --- | --- | --- |
| **28 day survival** | **S** | **S** | **NS** | **NS** | **NS** | **NS** | **S** | **S** | **S** | **S** | **NS** | **NS** | **S** | **S** | **S** | **NS** | **NS** | **S** | **NS** | **S** |
| **90 day survival** | **NS** | **S** | **NS** | **NS** | **NS** | **NS** | **S** | **NS** | **S** | **S** | **NS** | **NS** | **S** | **S** | **S** | **NS** | **NS** | **S** | **NS** | **S** |
| **LPC a C16:0** | 12.308 | 7.528 | 13.655 | 4.899 | 28.277 | 5.527 | 5.857 | 126.81 | 29.717 | 6.368 | 10.370 | 4.339 | 28.175 | 5.650 | 20.493 | 13.126 | 17.885 | 29.495 | 4.883 | 26.565 |
| **LPC a C16:1** | 0.467 | 0.310 | 0.587 | 0.304 | 0.604 | 0.250 | 0.308 | 3.733 | 0.900 | 0.315 | 0.305 | 0.313 | 1.213 | 0.390 | 0.972 | 0.643 | 0.514 | 0.966 | 0.244 | 0.657 |
| **LPC a C17:0** | 0.233 | 0.160 | 0.245 | 0.134 | 0.371 | 0.127 | 0.108 | 1.497 | 0.418 | 0.121 | 0.129 | 0.129 | 0.389 | 0.105 | 0.466 | 0.398 | 0.259 | 0.434 | 0.124 | 0.413 |
| **LPC a C18:0** | 2.965 | 1.698 | 3.869 | 1.810 | 4.715 | 1.546 | 1.933 | 27.255 | 11.051 | 1.620 | 1.944 | 1.607 | 6.423 | 1.501 | 6.753 | 2.707 | 4.094 | 7.320 | 1.311 | 4.364 |
| **LPC a C18:1** | 2.303 | 1.917 | 4.377 | 2.778 | 3.995 | 1.936 | 1.707 | 32.610 | 6.319 | 2.273 | 2.470 | 2.570 | 4.382 | 1.504 | 3.997 | 7.747 | 3.395 | 4.625 | 1.827 | 3.481 |
| **LPC a C18:2** | 1.591 | 1.716 | 3.163 | 3.908 | 3.172 | 1.509 | 1.432 | 18.028 | 3.972 | 1.454 | 1.807 | 2.068 | 3.218 | 1.438 | 2.005 | 9.460 | 2.774 | 2.922 | 1.163 | 2.186 |
| **LPC a C20:3** | 0.411 | 0.302 | 0.520 | 0.331 | 0.299 | 0.291 | 0.289 | 2.082 | 0.558 | 0.152 | 0.243 | 0.276 | 0.471 | 0.174 | 0.292 | 0.619 | 0.392 | 0.257 | 0.258 | 0.305 |
| **LPC a C20:4** | 0.663 | 0.700 | 1.741 | 0.799 | 1.194 | 0.577 | 0.535 | 5.442 | 1.584 | 0.596 | 0.473 | 0.657 | 2.074 | 0.544 | 1.104 | 1.881 | 1.038 | 1.252 | 0.316 | 2.752 |
| **LPC a C24:0** | 0.048 | 0.053 | 0.082 | 0.057 | 0.045 | 0.066 | 0.046 | 0.061 | 0.053 | 0.054 | 0.037 | 0.047 | 0.071 | 0.048 | 0.061 | 0.067 | 0.068 | 0.077 | 0.040 | 0.086 |
| **LPC a C28:1** | 0.095 | 0.162 | 0.102 | 0.124 | 0.090 | 0.153 | 0.094 | 0.062 | 0.061 | 0.078 | 0.083 | 0.072 | 0.190 | 0.063 | 0.103 | 0.106 | 0.101 | 0.119 | 0.077 | 0.099 |
| **PC aa C28:1** | 1.389 | 2.372 | 1.758 | 1.827 | 0.869 | 2.359 | 1.825 | 0.900 | 0.816 | 1.934 | 1.365 | 1.117 | 2.762 | 1.332 | 1.512 | 1.095 | 2.396 | 1.671 | 1.171 | 2.178 |
| **PC aa C30:0** | 2.389 | 3.149 | 2.706 | 3.148 | 1.256 | 3.640 | 5.623 | 1.167 | 1.693 | 3.472 | 2.373 | 2.814 | 2.980 | 2.887 | 2.058 | 1.453 | 3.100 | 2.226 | 3.411 | 2.643 |
| **PC aa C30:2** | 0.005 | 0.005 | 0.099 | 0.108 | 0.028 | 0.176 | 0.005 | 0.033 | 0.165 | 0.230 | 0.008 | 0.140 | 0.005 | 0.005 | 0.005 | 0.020 | 0.168 | 0.005 | 0.005 | 0.005 |
| **PC aa C32:0** | 125.63 | 145.25 | 152.50 | 201.59 | 77.721 | 339.95 | 366.3 | 83.603 | 103.32 | 189.38 | 155.58 | 235.21 | 97.08 | 134.58 | 139.87 | 104.56 | 263.13 | 145.31 | 217.59 | 195.19 |
| **PC aa C32:1** | 175.49 | 387.71 | 243.72 | 264.33 | 101.97 | 557.74 | 533.68 | 73.621 | 70.221 | 429.29 | 323.01 | 154.92 | 219.93 | 182.03 | 132.69 | 134.79 | 465.12 | 209.55 | 250.07 | 172.21 |
| **PC aa C32:2** | 18.334 | 22.073 | 19.708 | 19.061 | 12.173 | 37.801 | 32.218 | 6.500 | 12.095 | 39.112 | 23.987 | 9.939 | 30.387 | 16.521 | 11.947 | 18.256 | 22.541 | 27.910 | 12.636 | 19.743 |
| **PC aa C32:3** | 1.673 | 2.732 | 2.250 | 2.391 | 1.479 | 4.049 | 3.210 | 1.984 | 1.012 | 3.571 | 1.484 | 1.075 | 2.983 | 1.737 | 2.629 | 2.030 | 1.827 | 2.228 | 0.964 | 1.986 |
| **PC aa C34:1** | 1584.8 | 2322.5 | 1804.6 | 2344.6 | 1056.3 | 4074.2 | 2217.3 | 1087.6 | 600.8 | 2545.8 | 2812.2 | 1013.5 | 1447.9 | 1164.4 | 1388.1 | 1624.9 | 2375.4 | 1775 | 1409.2 | 1661.9 |
| **PC aa C34:2** | 2349.6 | 3045.5 | 1704.7 | 3347.7 | 2149.6 | 4671.1 | 3375.5 | 1433.5 | 928.1 | 3276.5 | 3433.3 | 931.9 | 2392.6 | 1389.3 | 1287.6 | 2117 | 2797.5 | 3341.8 | 1325.1 | 2298 |
| **PC aa C34:3** | 51.173 | 98.632 | 48.719 | 75.752 | 33.495 | 120.78 | 83.246 | 38.054 | 23.517 | 100.05 | 64.923 | 26.346 | 74.940 | 47.897 | 47.652 | 65.334 | 64.763 | 83.148 | 38.526 | 48.659 |
| **PC aa C34:4** | 3.307 | 5.312 | 4.037 | 2.872 | 2.095 | 4.766 | 3.737 | 2.406 | 2.708 | 5.947 | 2.938 | 1.645 | 9.618 | 2.826 | 2.782 | 3.562 | 4.398 | 5.129 | 1.938 | 5.721 |
| **PC aa C36:0** | 1.486 | 4.641 | 5.288 | 7.027 | 2.357 | 4.430 | 5.752 | 4.437 | 4.251 | 6.877 | 4.446 | 5.199 | 5.451 | 2.891 | 4.766 | 3.062 | 6.544 | 8.008 | 1.715 | 4.045 |
| **PC aa C36:1** | 121.6 | 154.6 | 139.3 | 232.01 | 52.396 | 335.15 | 186.60 | 85.354 | 69.877 | 178.84 | 158.00 | 117.35 | 119.84 | 98.012 | 147.02 | 140.12 | 188.34 | 112.81 | 128.58 | 75.463 |
| **PC aa C36:2** | 603.85 | 721.80 | 584.86 | 1265.3 | 399.78 | 1459.6 | 1136.4 | 454.66 | 411.66 | 1016.2 | 855.12 | 519.71 | 638.13 | 405.03 | 550.31 | 512.62 | 804.17 | 931.95 | 464.08 | 401.14 |
| **PC aa C36:3** | 370.84 | 399.26 | 258.87 | 354.73 | 191.27 | 527.96 | 444.36 | 402.46 | 194.57 | 473.05 | 274.33 | 197.27 | 349.44 | 132.2 | 208.79 | 198.01 | 304.45 | 431.07 | 204.73 | 222 |
| **PC aa C36:4** | 493.08 | 850.02 | 587.42 | 461.56 | 378.87 | 748.64 | 494.9 | 391.95 | 289.18 | 827.64 | 480.13 | 204.40 | 1022.2 | 315.94 | 447.4 | 343.51 | 745.61 | 884.69 | 198.14 | 1476.7 |
| **PC aa C36:5** | 39.923 | 61.006 | 30.019 | 23.315 | 13.704 | 51.738 | 31.281 | 24.106 | 13.360 | 66.059 | 20.593 | 19.447 | 57.214 | 20.384 | 97.671 | 31.324 | 34.486 | 51.571 | 13.582 | 47.169 |
| **PC aa C36:6** | 1.407 | 2.466 | 1.920 | 1.698 | 0.795 | 2.575 | 1.938 | 1.173 | 1.140 | 2.425 | 1.245 | 0.884 | 3.024 | 1.474 | 2.110 | 1.356 | 1.717 | 2.343 | 0.662 | 1.682 |
| **PC aa C38:0** | 2.226 | 2.305 | 2.960 | 3.885 | 1.430 | 4.069 | 5.301 | 4.472 | 3.923 | 2.923 | 2.382 | 6.375 | 5.042 | 1.612 | 4.919 | 2.572 | 9.338 | 4.225 | 2.258 | 2.129 |
| **PC aa C38:1** | 0.820 | 0.525 | 1.216 | 1.844 | 1.001 | 2.315 | 0.810 | 0.141 | 1.098 | 0.975 | 0.633 | 1.025 | 1.674 | 0.716 | 0.490 | 0.910 | 1.579 | 0.757 | 0.813 | 0.256 |
| **PC aa C38:3** | 68.015 | 77.979 | 61.634 | 74.542 | 29.689 | 91.442 | 83.003 | 76.352 | 53.123 | 70.838 | 42.068 | 39.549 | 82.685 | 30.194 | 62.894 | 31.884 | 60.488 | 81.985 | 40.770 | 39.979 |
| **PC aa C38:4** | 158.71 | 225.15 | 222.29 | 207.45 | 85.147 | 272.42 | 205.39 | 142.02 | 149.82 | 277.66 | 125.64 | 110.57 | 293.81 | 110.77 | 190.25 | 111.68 | 221.36 | 309.24 | 74.429 | 333.7 |
| **PC aa C38:5** | 70.061 | 85.475 | 84.263 | 65.633 | 32.458 | 104.18 | 67.953 | 72.708 | 48.878 | 116.95 | 39.687 | 47.965 | 108.13 | 34.525 | 91.693 | 42.411 | 96.910 | 112.49 | 32.115 | 118.88 |
| **PC aa C38:6** | 124.29 | 151.25 | 130.43 | 107.44 | 42.870 | 179.09 | 164.02 | 159.75 | 97.381 | 165.71 | 92.206 | 47.896 | 201.79 | 60.326 | 176.61 | 85.280 | 160.48 | 216.08 | 48.033 | 166.68 |
| **PC aa C40:1** | 0.483 | 0.549 | 0.585 | 0.556 | 0.414 | 0.637 | 0.630 | 0.704 | 0.488 | 0.823 | 0.593 | 0.554 | 0.555 | 0.516 | 0.559 | 0.462 | 0.637 | 0.783 | 0.637 | 0.676 |
| **PC aa C40:2** | 0.451 | 0.614 | 0.749 | 0.498 | 0.500 | 0.616 | 0.654 | 0.911 | 0.457 | 0.588 | 0.423 | 0.769 | 0.587 | 0.467 | 0.532 | 0.571 | 0.698 | 0.618 | 0.555 | 0.303 |
| **PC aa C40:3** | 0.371 | 0.667 | 1.036 | 0.561 | 0.355 | 0.843 | 0.603 | 1.106 | 0.672 | 0.684 | 0.292 | 0.701 | 0.767 | 0.294 | 0.466 | 0.355 | 0.553 | 0.858 | 0.522 | 0.434 |
| **PC aa C40:4** | 2.958 | 3.269 | 4.572 | 4.441 | 1.420 | 4.827 | 3.809 | 4.647 | 4.624 | 3.622 | 2.116 | 3.674 | 5.610 | 1.462 | 2.481 | 2.008 | 7.131 | 4.343 | 2.457 | 3.699 |
| **PC aa C40:5** | 8.385 | 10.263 | 12.545 | 14.314 | 2.800 | 15.016 | 8.517 | 8.464 | 9.158 | 12.808 | 4.878 | 6.765 | 11.513 | 4.236 | 7.303 | 3.990 | 13.734 | 12.040 | 4.499 | 12.757 |
| **PC aa C40:6** | 26.427 | 32.802 | 32.229 | 34.639 | 6.075 | 44.442 | 47.902 | 35.123 | 30.557 | 36.449 | 17.527 | 16.328 | 37.586 | 15.448 | 56.784 | 20.068 | 34.306 | 50.493 | 12.272 | 29.166 |
| **PC aa C42:0** | 0.151 | 0.263 | 0.280 | 0.357 | 0.154 | 0.517 | 0.439 | 0.353 | 0.285 | 0.483 | 0.417 | 0.451 | 0.482 | 0.223 | 0.451 | 0.300 | 0.486 | 0.590 | 0.170 | 0.342 |
| **PC aa C42:1** | 0.178 | 0.177 | 0.217 | 0.221 | 0.189 | 0.459 | 0.408 | 0.302 | 0.247 | 0.229 | 0.181 | 0.255 | 0.253 | 0.166 | 0.188 | 0.157 | 0.248 | 0.269 | 0.173 | 0.135 |
| **PC aa C42:2** | 0.049 | 0.206 | 0.193 | 0.063 | 0.139 | 0.314 | 0.195 | 0.226 | 0.096 | 0.155 | 0.211 | 0.219 | 0.262 | 0.079 | 0.179 | 0.135 | 0.147 | 0.313 | 0.139 | 0.186 |
| **PC aa C42:4** | 0.153 | 0.170 | 0.239 | 0.130 | 0.084 | 0.215 | 0.268 | 0.491 | 0.128 | 0.272 | 0.219 | 0.191 | 0.162 | 0.117 | 0.161 | 0.093 | 0.186 | 0.206 | 0.109 | 0.177 |
| **PC aa C42:5** | 0.159 | 0.128 | 0.226 | 0.340 | 0.111 | 0.266 | 0.379 | 0.427 | 0.393 | 0.279 | 0.154 | 0.334 | 0.393 | 0.077 | 0.247 | 0.228 | 0.653 | 0.288 | 0.066 | 0.332 |
| **PC aa C42:6** | 0.188 | 0.237 | 0.226 | 0.258 | 0.273 | 0.621 | 0.246 | 0.465 | 0.362 | 0.397 | 0.266 | 0.403 | 0.620 | 0.221 | 0.428 | 0.373 | 0.533 | 0.519 | 0.296 | 0.380 |
| **PC ae C30:0** | 0.208 | 0.213 | 0.200 | 0.231 | 0.111 | 0.305 | 0.366 | 0.103 | 0.172 | 0.261 | 0.200 | 0.291 | 0.282 | 0.184 | 0.165 | 0.174 | 0.265 | 0.191 | 0.247 | 0.167 |
| **PC ae C30:1** | 0.048 | 0.021 | 0.068 | 0.110 | 0.016 | 0.190 | 0.198 | 0.036 | 0.137 | 0.132 | 0.087 | 0.088 | 0.143 | 0.040 | 0.016 | 0.022 | 0.043 | 0.165 | 0.030 | 0.016 |
| **PC ae C30:2** | 0.027 | 0.050 | 0.049 | 0.067 | 0.037 | 0.082 | 0.057 | 0.037 | 0.025 | 0.082 | 0.040 | 0.039 | 0.090 | 0.041 | 0.054 | 0.042 | 0.061 | 0.065 | 0.033 | 0.046 |
| **PC ae C32:1** | 14.280 | 17.536 | 16.370 | 16.047 | 8.658 | 44.269 | 38.957 | 13.577 | 19.899 | 24.500 | 22.312 | 22.393 | 19.308 | 13.037 | 12.768 | 12.146 | 28.848 | 20.119 | 19.215 | 11.951 |
| **PC ae C32:2** | 3.492 | 4.676 | 4.615 | 2.955 | 3.097 | 10.287 | 9.239 | 3.487 | 5.021 | 4.658 | 4.071 | 4.992 | 4.447 | 2.944 | 4.092 | 2.703 | 4.320 | 5.110 | 3.726 | 3.041 |
| **PC ae C34:0** | 6.010 | 8.938 | 9.172 | 11.232 | 3.288 | 13.467 | 13.169 | 3.831 | 4.944 | 8.602 | 6.521 | 8.239 | 7.454 | 6.799 | 7.193 | 4.333 | 9.530 | 6.781 | 11.044 | 8.493 |
| **PC ae C34:1** | 59.345 | 91.884 | 57.706 | 94.780 | 37.345 | 161.03 | 96.857 | 37.168 | 31.174 | 89.234 | 76.397 | 72.656 | 67.511 | 50.353 | 58.168 | 54.251 | 116.15 | 64.332 | 85.099 | 48.770 |
| **PC ae C34:2** | 30.843 | 39.549 | 36.643 | 42.661 | 27.443 | 79.058 | 51.112 | 23.379 | 26.421 | 52.360 | 37.443 | 31.261 | 55.551 | 26.123 | 27.954 | 32.540 | 41.329 | 50.719 | 33.998 | 26.470 |
| **PC ae C34:3** | 11.642 | 13.078 | 21.353 | 11.268 | 12.379 | 29.502 | 32.199 | 11.010 | 16.469 | 18.320 | 14.850 | 17.602 | 32.352 | 8.764 | 12.847 | 8.398 | 14.292 | 21.928 | 13.514 | 12.399 |
| **PC ae C36:0** | 4.992 | 4.635 | 5.793 | 5.768 | 1.775 | 7.515 | 7.033 | 3.120 | 3.523 | 5.868 | 5.956 | 2.877 | 3.632 | 7.036 | 4.830 | 3.720 | 3.085 | 5.994 | 4.265 | 3.338 |
| **PC ae C36:1** | 29.941 | 43.497 | 39.038 | 37.140 | 27.466 | 54.561 | 37.123 | 28.793 | 18.660 | 36.182 | 30.313 | 19.698 | 29.252 | 23.200 | 29.011 | 24.944 | 28.293 | 33.901 | 25.893 | 26.845 |
| **PC ae C36:2** | 43.487 | 64.015 | 35.759 | 52.477 | 33.030 | 77.027 | 50.009 | 25.175 | 20.270 | 58.283 | 38.212 | 29.291 | 42.687 | 23.138 | 36.213 | 34.134 | 41.595 | 50.694 | 32.622 | 29.728 |
| **PC ae C36:3** | 14.084 | 20.314 | 17.322 | 22.522 | 11.601 | 34.720 | 22.569 | 14.781 | 14.192 | 23.113 | 17.890 | 19.134 | 24.602 | 9.548 | 13.856 | 13.165 | 23.080 | 25.537 | 16.286 | 11.038 |
| **PC ae C36:4** | 24.844 | 23.849 | 38.104 | 41.962 | 21.971 | 45.786 | 36.338 | 35.296 | 41.119 | 37.012 | 29.261 | 52.329 | 75.743 | 16.416 | 25.940 | 23.133 | 117.05 | 53.210 | 24.699 | 41.916 |
| **PC ae C36:5** | 16.904 | 17.619 | 31.034 | 14.035 | 15.546 | 37.990 | 25.894 | 24.506 | 34.383 | 23.300 | 19.552 | 31.923 | 47.710 | 10.697 | 19.977 | 14.435 | 44.546 | 39.704 | 16.212 | 31.474 |
| **PC ae C38:0** | 2.358 | 2.619 | 2.225 | 2.290 | 1.075 | 2.875 | 2.633 | 2.977 | 1.364 | 3.277 | 1.470 | 1.085 | 4.119 | 1.370 | 2.857 | 1.712 | 2.167 | 3.970 | 1.137 | 2.600 |
| **PC ae C38:1** | 0.962 | 1.581 | 2.576 | 1.930 | 2.290 | 3.165 | 2.683 | 3.920 | 3.042 | 1.471 | 1.081 | 1.831 | 2.314 | 0.824 | 2.221 | 0.700 | 1.763 | 1.801 | 0.749 | 0.624 |
| **PC ae C38:2** | 3.398 | 3.793 | 5.813 | 4.104 | 3.602 | 7.418 | 4.425 | 9.470 | 4.498 | 3.191 | 2.567 | 3.679 | 4.231 | 2.388 | 3.472 | 2.330 | 3.586 | 3.782 | 3.108 | 1.618 |
| **PC ae C38:3** | 7.427 | 9.649 | 15.910 | 7.321 | 11.125 | 11.154 | 8.716 | 20.970 | 10.635 | 8.633 | 4.590 | 5.779 | 12.360 | 5.081 | 7.722 | 4.186 | 7.128 | 9.700 | 5.051 | 6.025 |
| **PC ae C38:4** | 18.290 | 24.382 | 23.727 | 23.153 | 12.506 | 26.568 | 19.971 | 21.008 | 21.815 | 26.491 | 16.909 | 21.605 | 35.245 | 11.415 | 18.894 | 14.348 | 39.869 | 29.861 | 14.514 | 31.591 |
| **PC ae C38:5** | 18.666 | 17.771 | 30.943 | 26.187 | 17.462 | 38.287 | 24.921 | 43.366 | 36.651 | 26.705 | 20.822 | 53.775 | 47.643 | 11.568 | 29.397 | 18.318 | 81.208 | 39.133 | 22.989 | 25.559 |
| **PC ae C38:6** | 6.289 | 6.977 | 10.131 | 10.361 | 4.427 | 14.132 | 12.927 | 10.553 | 11.348 | 10.549 | 6.521 | 13.899 | 17.990 | 5.176 | 12.950 | 6.433 | 18.704 | 14.710 | 5.726 | 9.082 |
| **PC ae C40:1** | 0.372 | 0.668 | 1.116 | 0.413 | 0.600 | 1.076 | 0.702 | 2.090 | 1.294 | 1.069 | 0.466 | 0.897 | 1.354 | 0.268 | 0.611 | 0.718 | 0.683 | 1.270 | 0.224 | 0.911 |
| **PC ae C40:2** | 1.462 | 1.823 | 1.760 | 1.540 | 1.467 | 1.922 | 2.044 | 2.920 | 1.353 | 1.576 | 1.397 | 1.162 | 2.108 | 1.054 | 2.467 | 1.355 | 1.032 | 1.775 | 1.088 | 1.699 |
| **PC ae C40:3** | 1.215 | 1.315 | 4.203 | 1.456 | 2.233 | 2.616 | 1.883 | 6.073 | 4.185 | 1.337 | 0.851 | 1.119 | 2.358 | 0.908 | 1.764 | 0.839 | 1.294 | 1.494 | 1.053 | 1.297 |
| **PC ae C40:4** | 1.633 | 1.730 | 3.998 | 2.296 | 1.748 | 3.000 | 2.259 | 5.218 | 3.742 | 2.479 | 1.925 | 2.597 | 4.118 | 1.363 | 2.013 | 1.980 | 3.277 | 3.357 | 1.595 | 2.621 |
| **PC ae C40:5** | 2.802 | 2.709 | 7.592 | 3.927 | 3.081 | 5.564 | 3.743 | 14.475 | 7.973 | 3.662 | 2.883 | 5.829 | 7.389 | 1.576 | 4.099 | 2.043 | 7.971 | 5.360 | 2.907 | 3.349 |
| **PC ae C40:6** | 3.953 | 4.262 | 4.566 | 5.080 | 1.558 | 6.282 | 5.172 | 5.118 | 4.665 | 4.614 | 2.439 | 5.469 | 6.248 | 2.615 | 7.153 | 3.116 | 9.964 | 7.388 | 2.695 | 4.177 |
| **PC ae C42:0** | 0.680 | 0.808 | 1.056 | 0.778 | 0.749 | 1.148 | 0.873 | 1.040 | 0.795 | 0.842 | 0.977 | 0.796 | 0.742 | 0.743 | 0.776 | 0.596 | 0.842 | 0.978 | 0.566 | 0.748 |
| **PC ae C42:1** | 0.381 | 0.533 | 0.773 | 0.519 | 0.344 | 0.647 | 0.516 | 0.762 | 0.556 | 0.499 | 0.353 | 0.531 | 0.686 | 0.251 | 0.504 | 0.354 | 0.581 | 0.712 | 0.341 | 0.546 |
| **PC ae C42:2** | 0.213 | 0.305 | 0.384 | 0.264 | 0.175 | 0.534 | 0.439 | 0.485 | 0.392 | 0.478 | 0.293 | 0.301 | 0.489 | 0.185 | 0.571 | 0.142 | 0.253 | 0.283 | 0.262 | 0.191 |
| **PC ae C42:3** | 0.288 | 0.258 | 0.573 | 0.357 | 0.531 | 0.691 | 0.479 | 1.689 | 0.797 | 0.693 | 0.495 | 0.442 | 0.651 | 0.296 | 0.488 | 0.323 | 0.551 | 0.704 | 0.276 | 0.690 |
| **PC ae C42:4** | 0.379 | 0.336 | 0.607 | 0.367 | 0.183 | 0.738 | 0.272 | 0.504 | 0.721 | 0.261 | 0.268 | 0.588 | 0.524 | 0.278 | 0.351 | 0.211 | 0.461 | 0.677 | 0.252 | 0.305 |
| **PC ae C42:5** | 1.138 | 0.618 | 1.954 | 0.982 | 0.695 | 1.685 | 1.364 | 3.349 | 2.265 | 1.146 | 0.902 | 1.959 | 2.178 | 0.834 | 1.353 | 0.953 | 1.818 | 1.839 | 0.974 | 1.216 |
| **PC ae C44:3** | 0.086 | 0.066 | 0.249 | 0.128 | 0.116 | 0.107 | 0.212 | 0.382 | 0.108 | 0.098 | 0.096 | 0.116 | 0.202 | 0.105 | 0.091 | 0.058 | 0.240 | 0.178 | 0.115 | 0.061 |
| **PC ae C44:4** | 0.287 | 0.106 | 0.148 | 0.052 | 0.089 | 0.221 | 0.121 | 0.423 | 0.187 | 0.153 | 0.171 | 0.123 | 0.168 | 0.086 | 0.195 | 0.098 | 0.140 | 0.148 | 0.093 | 0.184 |
| **PC ae C44:5** | 0.422 | 0.312 | 0.389 | 0.419 | 0.270 | 0.710 | 0.573 | 0.587 | 0.752 | 0.416 | 0.468 | 0.771 | 0.755 | 0.277 | 0.459 | 0.312 | 0.685 | 0.661 | 0.379 | 0.348 |
| **PC ae C44:6** | 0.298 | 0.310 | 0.408 | 0.271 | 0.186 | 0.803 | 0.462 | 0.478 | 0.709 | 0.418 | 0.289 | 0.637 | 0.579 | 0.251 | 0.520 | 0.266 | 0.577 | 1.041 | 0.403 | 0.247 |
| **Sugars** | 4430.6 | 3860.5 | 5173.6 | 5106.3 | 7982.6 | 5473.1 | 5216.2 | 7194.9 | 7265.3 | 6874.3 | 8156.6 | 6474.9 | 7770.9 | 4941.7 | 8043.4 | 6111.5 | 10401 | 6787.8 | 11334 | 8207.7 |
| **Ala** | 165 | 80.7 | 604 | 353 | 132 | 207 | 1080 | 405 | 579 | 213 | 217 | 713 | 193 | 171 | 351 | 208 | 304 | 214 | 908 | 527 |
| **Arg** | 73 | 36.5 | 8.2 | 77.3 | 51.8 | 37.3 | 179 | 98.7 | 26 | 62.4 | 60.7 | 155 | 76 | 28.7 | 132 | 38.3 | 112 | 79.7 | 343 | 62.4 |
| **Asn** | 45.2 | 30.8 | 2.11 | 60.8 | 27.9 | 41.6 | 190 | 74.6 | 104 | 44 | 4.67 | 194 | 36.3 | 19.6 | 32.1 | 47.1 | 77.8 | 51.4 | 78.7 | 50.2 |
| **Asp** | 7.7 | 10.9 | 11.4 | 9.22 | 12.2 | 13.9 | 50.1 | 14.5 | 30.7 | 8 | 34.1 | 15.5 | 11.3 | 7.96 | 13.9 | 5.63 | 6.19 | 5.98 | 44.3 | 24.4 |
| **Cit** | 17.2 | 3.67 | 8.38 | 12.3 | 3.42 | 10.8 | 33 | 29.1 | 40.5 | 3.92 | 5.06 | 55.4 | 5.07 | 5.12 | 4.58 | 7.64 | 15.2 | 9.52 | 45.8 | 3.92 |
| **Gln** | 716 | 293 | 427 | 822 | 22.1 | 775 | 2390 | 853 | 544 | 429 | 283 | 2050 | 389 | 156 | 429 | 463 | 1230 | 371 | 1580 | 413 |
| **Glu** | 31.1 | 21.7 | 163 | 16.8 | 250 | 61.8 | 146 | 109 | 846 | 22.5 | 74.9 | 69 | 150 | 27.6 | 159 | 20 | 99.2 | 15.7 | 78.2 | 36.2 |
| **Gly** | 155 | 83.9 | 250 | 171 | 156 | 212 | 722 | 245 | 575 | 135 | 147 | 888 | 182 | 148 | 368 | 136 | 183 | 138 | 868 | 204 |
| **His** | 105 | 56.6 | 120 | 170 | 54.3 | 97.6 | 353 | 112 | 139 | 110 | 86.2 | 225 | 145 | 79.1 | 134 | 99.1 | 115 | 70.4 | 213 | 98.2 |
| **Ile** | 62.6 | 36.2 | 57.9 | 70.2 | 62.4 | 52.4 | 145 | 151 | 190 | 58.1 | 122 | 76.4 | 58.9 | 30 | 98.4 | 42.1 | 92.1 | 73.6 | 184 | 48.6 |
| **Leu** | 130 | 77 | 129 | 135 | 120 | 116 | 267 | 280 | 316 | 133 | 235 | 123 | 93.3 | 68.6 | 156 | 99.6 | 204 | 159 | 318 | 126 |
| **Lys** | 291 | 160 | 277 | 411 | 212 | 131 | 764 | 472 | 1060 | 237 | 261 | 766 | 215 | 114 | 300 | 158 | 311 | 257 | 613 | 276 |
| **Met** | 14.7 | 13.4 | 45.9 | 37.8 | 20.1 | 25.9 | 175 | 56.4 | 170 | 23.9 | 23 | 392 | 12.6 | 12.2 | 46.1 | 19.1 | 35.3 | 20.6 | 204 | 40.9 |
| **Orn** | 83.3 | 27 | 155 | 63.1 | 40.8 | 57 | 237 | 126 | 485 | 50.2 | 64.5 | 166 | 41.9 | 31.5 | 214 | 24.3 | 70.8 | 79.3 | 344 | 74.1 |
| **Phe** | 105 | 64 | 206 | 129 | 101 | 112 | 339 | 139 | 386 | 213 | 128 | 289 | 166 | 289 | 236 | 119 | 178 | 103 | 442 | 113 |
| **Pro** | 114 | 50.7 | 169 | 228 | 92 | 128 | 14 | 271 | 554 | 110 | 75.2 | 1050 | 87 | 47.4 | 201 | 61.3 | 369 | 78.5 | 704 | 114 |
| **Ser** | 37.8 | 38.3 | 64.3 | 35.2 | 82.3 | 46 | 161 | 155 | 145 | 34.1 | 53 | 100 | 35.8 | 32.7 | 116 | 33 | 73.9 | 37.1 | 190 | 71.7 |
| **Thr** | 50 | 41.1 | 120 | 76.8 | 52.6 | 55.3 | 3 | 180 | 236 | 62.3 | 54.5 | 335 | 73.6 | 39 | 110 | 52.4 | 99.3 | 77.3 | 213 | 105 |
| **Trp** | 19.5 | 14.9 | 55.8 | 16.5 | 29.1 | 10.7 | 45.1 | 114 | 195 | 26.5 | 24.5 | 40.4 | 10.8 | 8.320 | 82.4 | 9.490 | 13.7 | 27.9 | 76 | 47.2 |
| **Tyr** | 50.2 | 29.7 | 97.1 | 72.7 | 79.8 | 77.1 | 179 | 187 | 343 | 135 | 46.5 | 239 | 43 | 25.3 | 65.6 | 47.5 | 138 | 60.2 | 159 | 87.6 |
| **Val** | 189 | 123 | 229 | 222 | 199 | 181 | 425 | 531 | 559 | 261 | 518 | 260 | 207 | 141 | 403 | 224 | 366 | 333 | 941 | 256 |
| **Ac Orn** | 0.672 | 0.086 | 0.919 | 0.378 | 0.105 | 0.339 | 1.470 | 0.367 | 0.689 | 0.559 | 0.442 | 1.260 | 0.168 | 0.240 | 0.635 | 0.250 | 0.387 | 0.568 | 0.513 | 0.305 |
| **ADMA** | 0.503 | 0.147 | 0.210 | 0.436 | 0.402 | 0.542 | 1.290 | 0.633 | 2.770 | 0.800 | 0.411 | 2.400 | 0.561 | 0.332 | 0.528 | 0.485 | 1.820 | 0.591 | 1.950 | 0.437 |
| **Alpha AAA** | 1.510 | 0.564 | 3.130 | 1.120 | 1.410 | 1.480 | 7.630 | 1.520 | 5.460 | 0.708 | 0.673 | 1.900 | 1.030 | 0.719 | 0.924 | 1.410 | 0.545 | 1.420 | 1.180 | 0.896 |
| **Creatinine** | 230 | 38.200 | 329 | 142 | 51.8 | 231 | 250 | 87.5 | 172 | 274 | 194 | 315 | 418 | 276 | 215 | 114 | 140 | 289 | 117 | 118 |
| **DOPA** | 0.220 | 0.040 | 0.442 | 0.146 | 0.040 | 0.131 | 0.492 | 0.206 | 0.342 | 0.173 | 0.105 | 0.338 | 0.040 | 0.114 | 0.203 | 0.118 | 0.218 | 0.136 | 0.481 | 0.295 |
| **Kynurenine** | 5.880 | 2.390 | 19 | 8.660 | 5.680 | 7.440 | 13.8 | 5.8 | 20.3 | 10.2 | 8.730 | 13.4 | 27 | 21.3 | 12.3 | 13.5 | 21.2 | 12.7 | 14.4 | 7.51 |
| **Met SO** | 1.070 | 0.605 | 8.360 | 1.780 | 1.420 | 2.060 | 9.170 | 2.060 | 17.700 | 1.390 | 1.870 | 10.400 | 3.030 | 2.030 | 5.080 | 0.631 | 3.530 | 1.550 | 6.900 | 1.430 |
| **Putrescine** | 0.089 | 0.100 | 0.781 | 0.152 | 0.285 | 0.015 | 0.708 | 0.015 | 0.015 | 0.148 | 0.289 | 0.519 | 0.146 | 0.109 | 1.360 | 0.015 | 0.146 | 0.128 | 0.175 | 0.335 |
| **SDMA** | 1.880 | 0.541 | 2.330 | 1.070 | 0.150 | 1.470 | 1.550 | 0.996 | 2.490 | 1.890 | 1.270 | 3.620 | 1.170 | 1.560 | 3.570 | 0.708 | 1.920 | 3.220 | 1.590 | 0.652 |
| **Spermidine** | 0.076 | 0.126 | 0.110 | 0.031 | 0.058 | 0.035 | 0.203 | 0.072 | 0.116 | 0.047 | 0.038 | 0.076 | 0.041 | 0.037 | 0.103 | 0.107 | 0.031 | 0.045 | 0.109 | 0.104 |
| **Spermine** | 0.060 | 0.090 | 0.049 | 0.018 | 0.023 | 0.058 | 0.122 | 0.028 | 0.027 | 0.044 | 0.029 | 0.032 | 0.043 | 0.017 | 0.020 | 0.037 | 0.024 | 0.034 | 0.064 | 0.027 |
| **T4 OH Pro** | 5.620 | 2.320 | 7.610 | 13.2 | 20.4 | 15.4 | 97.8 | 8.9 | 58.9 | 7.970 | 17.4 | 118 | 10.8 | 5.08 | 5.41 | 6.09 | 15.1 | 17.5 | 32 | 13 |
| **Taurine** | 45.2 | 32.5 | 236 | 37.3 | 29.6 | 89.8 | 302 | 50.1 | 54.2 | 11.9 | 9.41 | 17.7 | 15.9 | 12.3 | 23.8 | 16.1 | 11.3 | 13.4 | 44 | 84.3 |
| **Total DMA** | 2.340 | 0.579 | 2.790 | 1.380 | 0.788 | 1.610 | 2.000 | 1.080 | 4.080 | 2.480 | 1.500 | 5.660 | 1.260 | 1.720 | 3.290 | 0.902 | 2.930 | 3.180 | 2.600 | 1.050 |
| **SM OH C14:1** | 1.927 | 2.896 | 2.265 | 2.783 | 1.496 | 3.068 | 2.190 | 1.383 | 0.899 | 2.222 | 1.842 | 1.350 | 3.277 | 1.898 | 2.451 | 1.530 | 3.296 | 2.218 | 1.772 | 2.466 |
| **SM OH C16:1** | 0.879 | 0.958 | 0.833 | 0.990 | 0.674 | 1.138 | 0.736 | 0.709 | 0.415 | 0.744 | 0.521 | 0.414 | 1.207 | 0.611 | 1.026 | 0.536 | 0.893 | 0.722 | 0.482 | 1.002 |
| **SM OH C22:1** | 0.317 | 0.375 | 0.571 | 0.426 | 0.225 | 0.444 | 0.399 | 0.473 | 0.315 | 0.382 | 0.330 | 0.210 | 0.930 | 0.380 | 0.432 | 0.297 | 0.388 | 0.521 | 0.259 | 0.471 |
| **SM OH C22:2** | 0.323 | 0.289 | 0.494 | 0.452 | 0.217 | 0.454 | 0.434 | 0.421 | 0.313 | 0.445 | 0.384 | 0.232 | 0.938 | 0.283 | 0.638 | 0.309 | 0.391 | 0.626 | 0.248 | 0.506 |
| **SM OH C24:1** | 0.024 | 0.006 | 0.036 | 0.031 | 0.017 | 0.044 | 0.053 | 0.036 | 0.025 | 0.033 | 0.017 | 0.018 | 0.061 | 0.021 | 0.053 | 0.012 | 0.035 | 0.033 | 0.052 | 0.023 |
| **SM C16:0** | 26.732 | 35.687 | 37.148 | 38.679 | 31.029 | 57.247 | 38.603 | 41.224 | 28.152 | 33.262 | 31.637 | 27.446 | 51.885 | 27.100 | 41.391 | 23.353 | 92.879 | 37.212 | 28.322 | 32.106 |
| **SM C16:1** | 3.268 | 5.583 | 5.135 | 6.245 | 4.346 | 9.005 | 6.496 | 6.564 | 3.909 | 7.046 | 5.535 | 4.926 | 9.491 | 4.257 | 7.438 | 3.720 | 11.318 | 6.502 | 3.235 | 5.237 |
| **SM C18:0** | 5.202 | 5.229 | 5.850 | 4.941 | 5.374 | 8.193 | 5.590 | 6.449 | 2.552 | 4.407 | 3.413 | 1.540 | 7.013 | 2.924 | 5.871 | 2.588 | 5.014 | 4.092 | 2.598 | 6.146 |
| **SM C18:1** | 1.819 | 2.395 | 2.654 | 2.579 | 2.134 | 4.023 | 2.357 | 3.419 | 1.221 | 3.175 | 1.856 | 0.862 | 3.773 | 1.317 | 3.600 | 1.345 | 2.242 | 2.521 | 0.880 | 2.856 |
| **SM C20:2** | 0.068 | 0.117 | 0.067 | 0.113 | 0.055 | 0.132 | 0.136 | 0.096 | 0.058 | 0.095 | 0.046 | 0.041 | 0.071 | 0.063 | 0.161 | 0.044 | 0.080 | 0.123 | 0.036 | 0.074 |
| **SM C22:3** | 0.012 | 0.005 | 0.045 | 0.118 | 0.059 | 0.074 | 0.058 | 0.036 | 0.035 | 0.005 | 0.005 | 0.005 | 0.005 | 0.023 | 0.005 | 0.025 | 0.099 | 0.036 | 0.005 | 0.014 |
| **SM C24:0** | 0.354 | 0.531 | 0.810 | 0.539 | 0.396 | 0.572 | 0.614 | 0.742 | 0.551 | 0.604 | 0.471 | 0.437 | 1.188 | 0.396 | 0.599 | 0.292 | 0.611 | 0.796 | 0.460 | 0.572 |
| **SM C24:1** | 1.303 | 1.356 | 2.197 | 1.577 | 2.483 | 2.490 | 2.052 | 2.997 | 1.640 | 1.522 | 1.865 | 1.513 | 2.939 | 1.313 | 3.382 | 1.129 | 2.434 | 2.133 | 1.650 | 1.740 |
| **SM C26:1** | 0.008 | 0.002 | 0.002 | 0.005 | 0.016 | 0.014 | 0.013 | 0.003 | 0.007 | 0.005 | 0.010 | 0.008 | 0.014 | 0.006 | 0.019 | 0.008 | 0.016 | 0.002 | 0.002 | 0.009 |
| **C0** | 36.538 | 8.516 | 91.795 | 24.626 | 37.951 | 55.467 | 90.904 | 55.998 | 91.804 | 30.205 | 50.097 | 71.447 | 27.299 | 26.090 | 66.774 | 34.947 | 58.050 | 32.785 | 75.781 | 25.816 |
| **C2** | 7.226 | 1.974 | 11.066 | 9.233 | 4.046 | 25.234 | 39.580 | 3.337 | 9.086 | 17.874 | 16.299 | 33.723 | 3.021 | 3.895 | 5.080 | 6.099 | 20.724 | 7.839 | 8.886 | 3.464 |
| **C4** | 0.330 | 0.173 | 0.789 | 0.364 | 0.271 | 0.676 | 0.878 | 0.325 | 0.239 | 0.336 | 0.740 | 0.424 | 0.222 | 0.178 | 0.507 | 0.200 | 0.660 | 0.574 | 0.720 | 0.180 |

**Table S4**. Plasma concentration (µM) of quantified metabolites for each patient at day 7 and survival rate at 28 and 90 days (S, survivor; NS, non survivor)

| **Metabolite D7** | **#1** | **#2** | **#3** | **#4** | **#5** | **#6** | **#7** | **#8** | **#9** | **#10** | **#11** | **#12** | **#13** | **#14** | **#15** | **#16** | **#17** | **#18** | **#19** | **#20** |
| --- | --- | --- | --- | --- | --- | --- | --- | --- | --- | --- | --- | --- | --- | --- | --- | --- | --- | --- | --- | --- |
| **28 day survival** | **S** | **S** | **NS** | **NS** | **NS** | **NS** | **S** | **S** | **S** | **S** | **NS** | **NS** | **S** | **S** | **S** | **NS** | **NS** | **S** | **NS** | **S** |
| **90 day survival** | **NS** | **S** | **NS** | **NS** | **NS** | **NS** | **S** | **NS** | **S** | **S** | **NS** | **NS** | **S** | **S** | **S** | **NS** | **NS** | **S** | **NS** | **S** |
| **LPC a C16:1** | 2.750 | 2.507 | 1.121 | 0.576 | 1.550 | 4.000 | 2.369 | 0.494 | 2.327 | 3.721 | 1.741 | 1.256 | 3.004 | 1.378 | 2.926 | 0.936 | 0.762 | 2.258 | 0.630 | 0.858 |
| **LPC a C17:0** | 1.210 | 1.013 | 0.340 | 0.269 | 0.339 | 0.850 | 0.787 | 0.131 | 0.508 | 1.006 | 0.488 | 0.279 | 0.697 | 0.222 | 0.709 | 0.534 | 0.293 | 1.122 | 0.130 | 0.442 |
| **LPC a C18:0** | 12.317 | 9.893 | 4.924 | 5.684 | 6.594 | 10.404 | 10.807 | 3.342 | 8.825 | 15.960 | 7.721 | 3.080 | 14.744 | 2.346 | 11.795 | 6.908 | 3.642 | 17.854 | 1.409 | 5.309 |
| **LPC a C18:1** | 17.099 | 20.421 | 10.772 | 7.772 | 12.801 | 30.973 | 13.072 | 5.807 | 16.064 | 27.513 | 12.215 | 7.810 | 10.495 | 3.109 | 14.485 | 15.905 | 4.872 | 19.699 | 5.000 | 5.600 |
| **LPC a C18:2** | 10.566 | 19.960 | 8.192 | 6.823 | 7.092 | 22.995 | 8.158 | 5.064 | 17.805 | 15.080 | 3.789 | 5.868 | 5.436 | 1.683 | 7.052 | 21.953 | 3.256 | 11.406 | 5.355 | 5.641 |
| **LPC a C20:3** | 1.493 | 1.338 | 0.872 | 0.406 | 1.316 | 3.745 | 1.235 | 0.267 | 0.746 | 2.113 | 0.955 | 0.367 | 0.999 | 0.382 | 1.008 | 0.989 | 0.490 | 1.424 | 0.612 | 0.486 |
| **LPC a C20:4** | 3.671 | 2.916 | 1.606 | 0.978 | 2.973 | 5.801 | 2.011 | 0.667 | 2.068 | 4.438 | 1.240 | 0.967 | 2.873 | 0.898 | 2.059 | 2.404 | 0.807 | 3.307 | 0.585 | 1.515 |
| **LPC a C24:0** | 0.093 | 0.104 | 0.080 | 0.085 | 0.066 | 0.096 | 0.142 | 0.075 | 0.102 | 0.093 | 0.060 | 0.086 | 0.096 | 0.064 | 0.109 | 0.062 | 0.066 | 0.120 | 0.041 | 0.084 |
| **LPC a C28:1** | 0.143 | 0.141 | 0.080 | 0.123 | 0.076 | 0.110 | 0.135 | 0.085 | 0.063 | 0.122 | 0.090 | 0.160 | 0.203 | 0.149 | 0.139 | 0.070 | 0.116 | 0.110 | 0.072 | 0.138 |
| **PC aa C28:1** | 1.907 | 2.100 | 1.487 | 1.955 | 0.847 | 1.709 | 1.579 | 1.118 | 1.183 | 2.330 | 1.567 | 2.736 | 2.781 | 1.852 | 1.746 | 1.435 | 2.332 | 1.635 | 0.748 | 1.794 |
| **PC aa C30:0** | 3.132 | 4.081 | 2.850 | 4.462 | 1.871 | 3.080 | 4.676 | 2.781 | 3.208 | 3.987 | 3.277 | 7.661 | 5.166 | 4.296 | 3.135 | 1.493 | 4.093 | 2.901 | 2.959 | 4.051 |
| **PC aa C30:2** | 0.005 | 0.005 | 0.037 | 0.100 | 0.005 | 0.059 | 0.070 | 0.060 | 0.074 | 0.005 | 0.274 | 0.356 | 0.005 | 0.215 | 0.247 | 0.047 | 0.122 | 0.005 | 0.083 | 0.119 |
| **PC aa C32:0** | 168.78 | 187.16 | 142.05 | 331.74 | 96.986 | 168.03 | 268.98 | 207.7 | 164.15 | 173.83 | 188.01 | 451.81 | 201.3 | 134.24 | 154.36 | 132.2 | 404.2 | 170.8 | 188.16 | 206.51 |
| **PC aa C32:1** | 249.77 | 266.14 | 194.71 | 287.7 | 144.29 | 416.55 | 395.58 | 174.32 | 162.21 | 268.43 | 343.82 | 435.23 | 382.44 | 572.41 | 389.91 | 101.31 | 774.17 | 159.55 | 290.92 | 230.62 |
| **PC aa C32:2** | 23.973 | 49.502 | 11.919 | 24.009 | 12.084 | 42.205 | 43.367 | 17.235 | 15.876 | 22.052 | 17.212 | 28.395 | 40.072 | 53.781 | 25.310 | 16.069 | 39.420 | 30.674 | 13.696 | 36.692 |
| **PC aa C32:3** | 2.694 | 4.149 | 1.432 | 2.603 | 2.206 | 3.896 | 4.271 | 1.442 | 1.310 | 3.482 | 1.932 | 1.965 | 5.785 | 5.123 | 3.486 | 2.447 | 2.019 | 3.830 | 1.049 | 2.992 |
| **PC aa C34:1** | 2036 | 2314.4 | 1562.4 | 3559.7 | 1233.6 | 2870.3 | 2695.1 | 2038.2 | 865.9 | 2356.5 | 2990.5 | 1478.8 | 2322.9 | 1353.2 | 2319.5 | 2022.3 | 3740.3 | 1923.7 | 1160.9 | 2088.5 |
| **PC aa C34:2** | 2141.4 | 3034.9 | 1229.9 | 4325.2 | 781.9 | 2300.1 | 2686.5 | 2668.1 | 1327.7 | 1823.5 | 1877.5 | 1350.2 | 1899. 2 | 1187.6 | 1077 | 3458.1 | 3832.7 | 2564.1 | 1227.1 | 3205.1 |
| **PC aa C34:3** | 77.561 | 172.34 | 40.878 | 130.95 | 51.929 | 129.92 | 141.35 | 55.915 | 43.644 | 107.79 | 69.660 | 56.852 | 152.12 | 143.65 | 108.58 | 93.371 | 135.10 | 115.73 | 54.052 | 127.87 |
| **PC aa C34:4** | 6.528 | 12.192 | 2.446 | 5.464 | 4.618 | 11.793 | 9.174 | 2.801 | 2.849 | 7.929 | 3.804 | 4.150 | 14.307 | 12.474 | 8.734 | 3.944 | 6.489 | 8.492 | 2.341 | 8.604 |
| **PC aa C36:0** | 5.274 | 7.325 | 3.387 | 6.533 | 1.593 | 7.179 | 8.500 | 5.568 | 6.927 | 4.892 | 1.481 | 7.186 | 6.045 | 3.309 | 2.284 | 5.637 | 9.316 | 5.215 | 2.033 | 7.056 |
| **PC aa C36:1** | 148.52 | 167.98 | 182.47 | 478.68 | 154.35 | 347.03 | 268.44 | 224.96 | 89.821 | 230.5 | 248.23 | 169.76 | 342.52 | 134.22 | 468.42 | 196.72 | 268.91 | 193.79 | 91.290 | 231.59 |
| **PC aa C36:2** | 598.4 | 910.25 | 636.61 | 1763.2 | 340.08 | 837.16 | 845.83 | 899.11 | 525.52 | 761.24 | 664.54 | 674.84 | 661.02 | 334.49 | 643.28 | 973.42 | 1089.6 | 949.81 | 426.31 | 990.12 |
| **PC aa C36:3** | 496.14 | 781.71 | 287.62 | 513.29 | 277.40 | 508.53 | 512.38 | 260.44 | 289.29 | 563.62 | 548.31 | 383.83 | 501.06 | 304.02 | 373.16 | 433.00 | 602.14 | 663.95 | 407.22 | 380.54 |
| **PC aa C36:4** | 615.67 | 792.36 | 268.09 | 426.30 | 369.82 | 642.19 | 508.83 | 244.08 | 200.10 | 694.66 | 400.26 | 364.54 | 994.50 | 480.46 | 510.86 | 368.73 | 574.95 | 660.61 | 196.54 | 724.87 |
| **PC aa C36:5** | 52.972 | 84.406 | 25.797 | 28.331 | 32.121 | 135.12 | 52.253 | 18.540 | 23.250 | 117.47 | 21.721 | 36.503 | 105.54 | 77.650 | 113.74 | 48.427 | 39.509 | 66.158 | 19.275 | 48.539 |
| **PC aa C36:6** | 2.366 | 4.228 | 1.283 | 2.445 | 1.059 | 4.844 | 3.110 | 1.443 | 1.233 | 3.719 | 1.467 | 2.211 | 3.881 | 2.639 | 3.473 | 1.796 | 2.077 | 3.006 | 1.068 | 2.301 |
| **PC aa C38:0** | 5.829 | 3.538 | 2.781 | 5.435 | 1.896 | 7.118 | 9.661 | 4.170 | 4.567 | 4.847 | 3.715 | 10.055 | 4.631 | 3.387 | 6.338 | 4.617 | 10.230 | 7.088 | 2.121 | 4.277 |
| **PC aa C38:1** | 0.990 | 2.180 | 1.734 | 0.374 | 0.504 | 1.989 | 2.531 | 1.762 | 2.211 | 2.446 | 0.069 | 0.388 | 1.021 | 0.658 | 0.843 | 1.365 | 1.133 | 3.499 | 0.156 | 0.000 |
| **PC aa C38:3** | 90.577 | 104.07 | 59.051 | 87.135 | 84.848 | 115.97 | 109.53 | 38.895 | 47.935 | 111.18 | 101.82 | 71.647 | 151.6 | 71.059 | 127.39 | 46.071 | 82.570 | 115.50 | 50.772 | 74.177 |
| **PC aa C38:4** | 198.27 | 188.16 | 114.50 | 239.94 | 168.95 | 230.44 | 182.81 | 108.59 | 79.353 | 236.87 | 130.6 | 132.15 | 435.24 | 132.73 | 275.75 | 128.19 | 207.47 | 236.56 | 59.148 | 252.15 |
| **PC aa C38:5** | 116.96 | 118.82 | 44.280 | 76.642 | 64.701 | 143.42 | 84.406 | 36.125 | 38.712 | 137.42 | 48.996 | 46.907 | 185.41 | 53.837 | 132.48 | 54.638 | 104.12 | 132.02 | 27.424 | 90.264 |
| **PC aa C38:6** | 155.16 | 102.69 | 43.300 | 105.39 | 46.501 | 138.73 | 153.13 | 57.988 | 43.902 | 151.82 | 55.175 | 32.700 | 125.8 | 44.638 | 136.40 | 109.61 | 145.03 | 170.24 | 21.440 | 112.62 |
| **PC aa C40:1** | 0.675 | 0.601 | 0.543 | 0.783 | 0.474 | 0.536 | 0.935 | 0.676 | 0.726 | 0.669 | 0.563 | 0.752 | 0.671 | 0.722 | 0.681 | 0.422 | 0.834 | 0.664 | 0.486 | 0.780 |
| **PC aa C40:2** | 0.544 | 0.900 | 0.689 | 0.779 | 0.703 | 0.607 | 1.112 | 0.648 | 0.749 | 0.761 | 0.688 | 1.086 | 0.764 | 0.549 | 0.502 | 0.555 | 0.896 | 0.862 | 0.604 | 0.568 |
| **PC aa C40:3** | 0.698 | 0.839 | 0.846 | 1.353 | 0.573 | 0.719 | 1.461 | 0.599 | 0.714 | 0.874 | 0.830 | 1.360 | 1.290 | 0.568 | 0.601 | 0.746 | 1.765 | 1.224 | 0.809 | 0.897 |
| **PC aa C40:4** | 4.987 | 4.058 | 2.774 | 5.669 | 3.484 | 5.305 | 4.817 | 2.928 | 3.207 | 3.570 | 3.458 | 4.029 | 8.076 | 2.580 | 4.932 | 2.473 | 8.854 | 5.846 | 2.183 | 5.720 |
| **PC aa C40:5** | 12.230 | 8.047 | 5.240 | 13.712 | 6.644 | 14.921 | 10.595 | 5.515 | 5.005 | 11.011 | 4.466 | 6.140 | 18.502 | 5.225 | 13.741 | 6.375 | 15.472 | 14.081 | 3.757 | 13.377 |
| **PC aa C40:6** | 30.574 | 17.710 | 13.348 | 32.010 | 15.010 | 33.292 | 37.375 | 12.873 | 10.530 | 34.100 | 12.467 | 9.643 | 35.919 | 10.088 | 57.616 | 21.901 | 32.447 | 40.677 | 5.819 | 26.914 |
| **PC aa C42:0** | 0.414 | 0.431 | 0.226 | 0.564 | 0.156 | 0.645 | 0.778 | 0.232 | 0.439 | 0.450 | 0.260 | 0.574 | 0.493 | 0.445 | 0.449 | 0.415 | 0.552 | 0.787 | 0.246 | 0.447 |
| **PC aa C42:1** | 0.344 | 0.269 | 0.189 | 0.266 | 0.245 | 0.250 | 0.342 | 0.151 | 0.219 | 0.141 | 0.107 | 0.279 | 0.468 | 0.340 | 0.345 | 0.239 | 0.422 | 0.408 | 0.198 | 0.419 |
| **PC aa C42:2** | 0.194 | 0.222 | 0.177 | 0.238 | 0.123 | 0.213 | 0.286 | 0.302 | 0.323 | 0.153 | 0.215 | 0.371 | 0.278 | 0.148 | 0.205 | 0.291 | 0.227 | 0.267 | 0.185 | 0.207 |
| **PC aa C42:4** | 0.107 | 0.187 | 0.237 | 0.369 | 0.082 | 0.413 | 0.192 | 0.109 | 0.214 | 0.149 | 0.189 | 0.331 | 0.343 | 0.115 | 0.282 | 0.226 | 0.312 | 0.204 | 0.112 | 0.254 |
| **PC aa C42:5** | 0.314 | 0.336 | 0.233 | 0.316 | 0.238 | 0.186 | 0.250 | 0.301 | 0.175 | 0.305 | 0.183 | 0.500 | 0.413 | 0.174 | 0.312 | 0.304 | 0.570 | 0.316 | 0.245 | 0.451 |
| **PC aa C42:6** | 0.536 | 0.382 | 0.334 | 0.702 | 0.297 | 0.479 | 0.518 | 0.249 | 0.518 | 0.421 | 0.476 | 0.658 | 0.617 | 0.344 | 0.447 | 0.388 | 0.721 | 0.365 | 0.265 | 0.292 |
| **PC ae C30:0** | 0.283 | 0.301 | 0.251 | 0.475 | 0.130 | 0.254 | 0.417 | 0.173 | 0.251 | 0.374 | 0.276 | 0.907 | 0.288 | 0.366 | 0.220 | 0.147 | 0.312 | 0.293 | 0.214 | 0.323 |
| **PC ae C30:1** | 0.046 | 0.129 | 0.122 | 0.090 | 0.016 | 0.117 | 0.179 | 0.048 | 0.167 | 0.077 | 0.094 | 0.277 | 0.128 | 0.124 | 0.164 | 0.068 | 0.069 | 0.107 | 0.118 | 0.051 |
| **PC ae C30:2** | 0.064 | 0.069 | 0.044 | 0.070 | 0.032 | 0.077 | 0.077 | 0.045 | 0.035 | 0.073 | 0.050 | 0.088 | 0.077 | 0.054 | 0.071 | 0.053 | 0.082 | 0.064 | 0.026 | 0.058 |
| **PC ae C32:1** | 27.758 | 31.071 | 22.107 | 40.812 | 10.006 | 46.048 | 69.749 | 25.331 | 35.333 | 29.846 | 31.845 | 78.824 | 26.744 | 39.233 | 27.023 | 19.280 | 34.558 | 34.076 | 18.693 | 32.126 |
| **PC ae C32:2** | 6.148 | 7.687 | 5.637 | 5.919 | 2.707 | 10.543 | 10.880 | 4.654 | 7.867 | 6.342 | 5.572 | 14.615 | 8.596 | 7.878 | 6.659 | 3.877 | 5.099 | 7.789 | 3.720 | 4.859 |
| **PC ae C34:0** | 11.635 | 11.113 | 9.573 | 15.966 | 3.332 | 8.397 | 14.838 | 6.276 | 6.081 | 10.346 | 7.687 | 19.726 | 9.245 | 7.126 | 6.883 | 6.308 | 12.721 | 7.204 | 8.096 | 8.494 |
| **PC ae C34:1** | 102.18 | 94.683 | 66.171 | 144.63 | 47.508 | 128.69 | 150.75 | 91.301 | 58.008 | 99.144 | 108.74 | 161.01 | 91.964 | 98.187 | 99.800 | 66.571 | 151.39 | 91.570 | 75.032 | 77.242 |
| **PC ae C34:2** | 51.199 | 67.791 | 37.244 | 92.822 | 24.817 | 81.084 | 113.9 | 55.247 | 45.272 | 44.946 | 45.619 | 64.065 | 66.512 | 56.235 | 43.325 | 59.188 | 52.748 | 78.248 | 30.923 | 71.862 |
| **PC ae C34:3** | 20.539 | 28.189 | 15.776 | 19.233 | 12.902 | 34.449 | 42.278 | 20.304 | 26.979 | 16.739 | 18.108 | 29.641 | 43.992 | 27.981 | 17.480 | 18.013 | 16.625 | 32.086 | 10.666 | 20.068 |
| **PC ae C36:0** | 5.861 | 2.832 | 11.438 | 6.669 | 1.231 | 4.604 | 7.701 | 5.700 | 4.183 | 4.902 | 5.125 | 7.998 | 5.023 | 2.851 | 5.145 | 3.195 | 7.829 | 4.895 | 3.038 | 3.227 |
| **PC ae C36:1** | 39.346 | 46.451 | 37.504 | 50.540 | 18.046 | 51.228 | 53.240 | 35.814 | 21.423 | 37.752 | 32.784 | 36.172 | 36.215 | 25.897 | 47.728 | 30.269 | 41.492 | 34.413 | 19.077 | 35.152 |
| **PC ae C36:2** | 56.369 | 72.835 | 36.010 | 99.458 | 21.442 | 65.848 | 70.978 | 48.006 | 30.824 | 46.104 | 42.593 | 48.540 | 43.900 | 33.955 | 40.995 | 62.851 | 57.628 | 61.528 | 28.111 | 69.204 |
| **PC ae C36:3** | 28.730 | 32.186 | 17.286 | 42.817 | 14.228 | 40.225 | 59.529 | 34.816 | 21.126 | 27.248 | 30.503 | 27.852 | 31.531 | 30.411 | 25.208 | 31.540 | 32.656 | 43.501 | 18.177 | 26.169 |
| **PC ae C36:4** | 59.126 | 50.398 | 31.416 | 57.318 | 26.812 | 79.505 | 90.168 | 32.668 | 49.851 | 54.051 | 59.660 | 98.348 | 73.540 | 56.323 | 39.445 | 42.869 | 122.66 | 69.836 | 22.559 | 62.643 |
| **PC ae C36:5** | 40.009 | 38.045 | 24.566 | 20.751 | 17.208 | 65.108 | 46.751 | 19.331 | 46.209 | 28.810 | 23.337 | 53.097 | 46.500 | 40.898 | 25.442 | 24.236 | 50.443 | 52.237 | 13.609 | 38.827 |
| **PC ae C38:0** | 3.367 | 3.935 | 1.678 | 2.502 | 1.453 | 5.208 | 3.935 | 1.844 | 1.854 | 4.525 | 1.545 | 2.226 | 5.007 | 1.949 | 4.497 | 2.537 | 3.570 | 4.221 | 1.070 | 2.948 |
| **PC ae C38:1** | 1.364 | 1.778 | 3.907 | 3.183 | 1.487 | 2.947 | 3.543 | 5.329 | 2.542 | 1.263 | 1.734 | 3.153 | 1.754 | 1.573 | 2.049 | 1.553 | 2.663 | 2.610 | 1.117 | 0.911 |
| **PC ae C38:2** | 4.806 | 5.093 | 5.427 | 10.588 | 4.486 | 4.040 | 9.471 | 6.381 | 5.141 | 5.730 | 4.401 | 6.864 | 4.485 | 3.719 | 4.668 | 4.813 | 6.023 | 5.303 | 3.588 | 4.539 |
| **PC ae C38:3** | 11.448 | 12.501 | 10.770 | 11.813 | 9.606 | 12.678 | 19.965 | 9.020 | 9.312 | 12.499 | 10.032 | 9.492 | 11.620 | 10.706 | 11.580 | 8.094 | 8.552 | 13.361 | 5.636 | 8.251 |
| **PC ae C38:4** | 35.248 | 28.867 | 16.737 | 34.930 | 17.102 | 38.700 | 47.214 | 18.186 | 21.616 | 32.925 | 29.200 | 40.174 | 41.221 | 27.383 | 27.288 | 20.256 | 42.606 | 38.538 | 12.449 | 40.442 |
| **PC ae C38:5** | 59.291 | 41.913 | 27.454 | 47.061 | 30.036 | 70.104 | 74.883 | 33.758 | 53.642 | 46.380 | 47.890 | 99.991 | 52.357 | 41.273 | 43.650 | 41.137 | 97.221 | 67.743 | 23.467 | 42.471 |
| **PC ae C38:6** | 15.864 | 12.001 | 8.180 | 13.188 | 6.314 | 26.676 | 24.308 | 8.783 | 14.374 | 13.990 | 9.198 | 20.521 | 16.248 | 12.503 | 15.169 | 11.101 | 22.423 | 20.199 | 4.858 | 11.970 |
| **PC ae C40:1** | 1.337 | 1.836 | 0.643 | 0.836 | 0.844 | 1.559 | 1.695 | 1.101 | 1.404 | 1.669 | 0.812 | 1.210 | 1.795 | 0.660 | 1.710 | 1.031 | 1.288 | 1.633 | 0.585 | 1.166 |
| **PC ae C40:2** | 2.237 | 2.253 | 2.052 | 2.984 | 1.818 | 2.275 | 2.691 | 1.694 | 2.096 | 1.907 | 2.068 | 1.967 | 2.041 | 1.565 | 2.358 | 2.591 | 1.904 | 2.306 | 1.136 | 2.323 |
| **PC ae C40:3** | 1.884 | 1.631 | 3.211 | 1.901 | 2.566 | 2.315 | 4.784 | 3.206 | 2.746 | 2.026 | 1.925 | 1.820 | 2.355 | 2.119 | 2.259 | 1.170 | 1.898 | 2.524 | 1.160 | 2.023 |
| **PC ae C40:4** | 3.925 | 2.868 | 3.024 | 4.519 | 3.143 | 4.220 | 6.654 | 3.392 | 4.277 | 3.294 | 3.066 | 4.029 | 5.585 | 2.565 | 3.245 | 3.145 | 3.425 | 4.337 | 1.824 | 3.887 |
| **PC ae C40:5** | 6.674 | 4.457 | 4.444 | 6.877 | 4.109 | 8.650 | 11.727 | 6.461 | 6.929 | 6.300 | 5.472 | 11.282 | 8.114 | 4.467 | 6.447 | 4.328 | 9.410 | 10.243 | 2.422 | 6.705 |
| **PC ae C40:6** | 7.866 | 4.652 | 2.991 | 7.274 | 3.208 | 10.451 | 11.139 | 4.125 | 5.867 | 6.673 | 4.232 | 9.809 | 7.829 | 5.161 | 8.243 | 6.216 | 11.143 | 9.723 | 2.221 | 7.228 |
| **PC ae C42:0** | 0.910 | 0.779 | 0.809 | 0.893 | 0.697 | 0.953 | 1.084 | 0.685 | 0.619 | 0.895 | 0.652 | 0.967 | 0.853 | 0.585 | 0.782 | 0.669 | 1.191 | 1.154 | 0.750 | 0.731 |
| **PC ae C42:1** | 0.542 | 0.681 | 0.643 | 0.594 | 0.503 | 0.845 | 0.560 | 0.662 | 0.534 | 0.547 | 0.402 | 0.652 | 0.790 | 0.479 | 0.571 | 0.439 | 0.975 | 0.711 | 0.359 | 0.843 |
| **PC ae C42:2** | 0.450 | 0.408 | 0.275 | 0.277 | 0.233 | 0.352 | 0.827 | 0.390 | 0.276 | 0.495 | 0.340 | 0.611 | 0.458 | 0.300 | 0.523 | 0.320 | 0.501 | 0.393 | 0.226 | 0.485 |
| **PC ae C42:3** | 0.841 | 0.834 | 0.434 | 0.541 | 0.454 | 0.925 | 1.366 | 0.640 | 0.713 | 0.923 | 0.648 | 0.660 | 0.772 | 0.373 | 0.852 | 0.681 | 0.700 | 0.886 | 0.336 | 0.602 |
| **PC ae C42:4** | 0.826 | 0.408 | 0.498 | 0.650 | 0.455 | 0.913 | 1.439 | 0.773 | 1.127 | 0.594 | 0.600 | 0.669 | 0.749 | 0.656 | 0.914 | 0.661 | 0.608 | 0.828 | 0.241 | 0.788 |
| **PC ae C42:5** | 2.164 | 1.735 | 1.256 | 1.663 | 1.050 | 2.752 | 3.865 | 1.644 | 2.764 | 1.951 | 2.138 | 3.318 | 2.108 | 1.831 | 2.666 | 1.542 | 1.731 | 3.711 | 0.933 | 1.900 |
| **PC ae C44:3** | 0.171 | 0.133 | 0.054 | 0.202 | 0.141 | 0.180 | 0.152 | 0.131 | 0.126 | 0.120 | 0.117 | 0.141 | 0.157 | 0.108 | 0.060 | 0.158 | 0.166 | 0.174 | 0.122 | 0.076 |
| **PC ae C44:4** | 0.164 | 0.237 | 0.143 | 0.249 | 0.164 | 0.327 | 0.274 | 0.197 | 0.193 | 0.297 | 0.324 | 0.204 | 0.219 | 0.216 | 0.257 | 0.225 | 0.206 | 0.238 | 0.168 | 0.155 |
| **PC ae C44:5** | 0.974 | 0.574 | 0.411 | 0.411 | 0.277 | 1.733 | 1.444 | 0.537 | 1.160 | 0.701 | 0.704 | 1.523 | 0.782 | 0.598 | 0.707 | 0.815 | 0.694 | 0.858 | 0.436 | 0.720 |
| **PC ae C44:6** | 0.636 | 0.626 | 0.236 | 0.433 | 0.346 | 1.220 | 1.042 | 0.393 | 0.947 | 0.689 | 0.519 | 1.288 | 0.785 | 0.766 | 0.923 | 0.452 | 0.804 | 0.883 | 0.401 | 0.884 |
| **Sugars** | 8079.7 | 7240.2 | 4793.8 | 4408.4 | 7463.5 | 7087.4 | 8615.3 | 16726 | 5410.5 | 8425.9 | 8411.7 | 9153.4 | 7390.9 | 10431 | 7747.9 | 7737.1 | 6909 | 7399.8 | 11244 | 13247 |
| **Ala** | 228 | 230 | 360 | 484 | 139 | 484 | 527 | 696 | 573 | 325 | 272 | 1980 | 301 | 339 | 383 | 141 | 282 | 129 | 693 | 288 |
| **Arg** | 123 | 67.6 | 96.8 | 101 | 143 | 150 | 122 | 241 | 238 | 115 | 131 | 365 | 165 | 230 | 191 | 76.8 | 143 | 90.4 | 129 | 58.4 |
| **Asn** | 64.4 | 78 | 36.3 | 91.7 | 18.4 | 66.4 | 98.4 | 80.4 | 109 | 93.6 | 35.7 | 404 | 71.8 | 57.8 | 33.6 | 47 | 87.1 | 40.5 | 138 | 54 |
| **Asp** | 4.7 | 14.7 | 37.6 | 21.9 | 37 | 5.720 | 21.7 | 16.3 | 10.2 | 7.340 | 8.660 | 16.6 | 13 | 9.410 | 2.760 | 7.050 | 15.8 | 4.490 | 25.7 | 22.1 |
| **Cit** | 28.4 | 13.8 | 22.9 | 24 | 9.210 | 15.5 | 19 | 35 | 15.9 | 19.2 | 24.1 | 215 | 31.4 | 17.4 | 9.950 | 13 | 19.6 | 30.6 | 40.9 | 14.7 |
| **Gln** | 842 | 629 | 38.1 | 835 | 24.2 | 941 | 589 | 72.8 | 423 | 863 | 490 | 3120 | 516 | 492 | 742 | 650 | 787 | 517 | 1160 | 637 |
| **Glu** | 45.8 | 107 | 477 | 65.1 | 310 | 46.5 | 272 | 594 | 307 | 67.3 | 152 | 67.8 | 349 | 268 | 55.2 | 57 | 262 | 98.6 | 97.1 | 85.9 |
| **Gly** | 237 | 287 | 253 | 303 | 183 | 385 | 380 | 520 | 327 | 274 | 341 | 1830 | 202 | 473 | 525 | 157 | 297 | 187 | 344 | 216 |
| **His** | 73.2 | 62 | 118 | 154 | 62.8 | 118 | 126 | 109 | 122 | 96 | 75.2 | 235 | 124 | 115 | 108 | 53.8 | 109 | 60.5 | 169 | 72.5 |
| **Ile** | 102 | 52.8 | 55.1 | 94.3 | 98.3 | 76 | 80.1 | 88.5 | 130 | 76.2 | 73.1 | 202 | 107 | 87 | 111 | 62.7 | 52.4 | 49.1 | 137 | 64.8 |
| **Leu** | 221 | 116 | 108 | 144 | 123 | 147 | 170 | 178 | 226 | 173 | 107 | 358 | 222 | 163 | 160 | 123 | 113 | 114 | 287 | 139 |
| **Lys** | 365 | 328 | 287 | 344 | 318 | 430 | 486 | 608 | 714 | 380 | 252 | 1440 | 595 | 703 | 425 | 273 | 317 | 333 | 511 | 370 |
| **Met** | 33.7 | 36.7 | 38.4 | 1110 | 60.5 | 56.7 | 70.2 | 857 | 760 | 35.8 | 35.1 | 577 | 39.8 | 62.2 | 62.8 | 24.5 | 38 | 19.9 | 88.9 | 35.3 |
| **Orn** | 176 | 111 | 62.8 | 121 | 98.8 | 97.6 | 202 | 177 | 231 | 92.1 | 141 | 486 | 141 | 149 | 237 | 103 | 74 | 112 | 164 | 181 |
| **Phe** | 145 | 111 | 196 | 130 | 178 | 184 | 218 | 240 | 122 | 174 | 176 | 392 | 128 | 158 | 193 | 166 | 145 | 114 | 303 | 197 |
| **Pro** | 219 | 308 | 174 | 346 | 47.9 | 246 | 247 | 403 | 321 | 201 | 181 | 1310 | 336 | 268 | 316 | 139 | 325 | 235 | 371 | 147 |
| **Ser** | 81.7 | 110 | 90.1 | 96.6 | 48.2 | 112 | 172 | 233 | 110 | 104 | 110 | 408 | 147 | 164 | 155 | 81.1 | 97.9 | 54.4 | 112 | 109 |
| **Thr** | 84.4 | 131 | 137 | 212 | 167 | 135 | 152 | 230 | 213 | 201 | 113 | 739 | 228 | 343 | 118 | 139 | 125 | 105 | 195 | 140 |
| **Trp** | 51.7 | 52.9 | 54.7 | 59.6 | 78.4 | 63.4 | 130 | 1 | 120 | 86.1 | 67.1 | 139 | 84 | 70.5 | 94.9 | 29.3 | 50.5 | 53.7 | 101 | 82.1 |
| **Tyr** | 83.9 | 78.5 | 127 | 111 | 55.8 | 88 | 187 | 170 | 294 | 117 | 65.6 | 541 | 115 | 114 | 92.6 | 72.6 | 157 | 84.8 | 289 | 121 |
| **Val** | 376 | 297 | 210 | 392 | 423 | 249 | 311 | 328 | 567 | 353 | 263 | 638 | 481 | 336 | 383 | 285 | 260 | 273 | 689 | 297 |
| **Ac Orn** | 1.690 | 0.392 | 0.082 | 0.194 | 0.075 | 0.157 | 0.780 | 0.273 | 0.042 | 0.174 | 0.278 | 1.380 | 0.359 | 0.241 | 0.425 | 0.285 | 0.476 | 0.712 | 0.829 | 0.161 |
| **ADMA** | 0.710 | 0.722 | 0.863 | 1.020 | 0.520 | 0.858 | 0.954 | 0.622 | 1.420 | 0.661 | 1.090 | 3.020 | 0.984 | 1.000 | 0.758 | 0.581 | 1.630 | 1.050 | 1.680 | 0.762 |
| **Alpha AAA** | 1.460 | 2.300 | 1.410 | 5.400 | 2.090 | 1.230 | 2.380 | 1.810 | 2.830 | 1.260 | 1.130 | 6.490 | 3.160 | 2.600 | 1.170 | 1.740 | 0.150 | 2.300 | 0.989 | 2.840 |
| **Creatinine** | 178 | 43.7 | 295 | 156 | 48.7 | 64.8 | 74.9 | 72.7 | 56.3 | 143 | 137 | 185 | 517 | 72.3 | 109 | 145 | 583 | 175 | 188 | 120 |
| **DOPA** | 0.155 | 0.205 | 0.174 | 0.212 | 0.140 | 0.236 | 0.438 | 0.434 | 0.399 | 0.203 | 0.176 | 0.583 | 0.201 | 0.230 | 0.231 | 0.132 | 0.040 | 0.092 | 0.356 | 0.223 |
| **Kynurenine** | 6.580 | 2.280 | 12.000 | 33.400 | 9.280 | 7.140 | 5.580 | 7.680 | 3.820 | 9.470 | 6.660 | 20.600 | 8.210 | 12.300 | 8.910 | 11.600 | 45.000 | 8.160 | 16.800 | 4.760 |
| **Met:SO** | 0.972 | 2.420 | 5.530 | 17.500 | 2.580 | 2.040 | 2.950 | 50.200 | 20.900 | 2.460 | 2.410 | 15.100 | 5.300 | 8.200 | 3.570 | 1.970 | 3.430 | 1.690 | 5.680 | 0.833 |
| **Putrescine** | 0.270 | 0.273 | 2.350 | 0.180 | 0.271 | 0.459 | 0.528 | 0.245 | 0.345 | 0.278 | 0.290 | 1.440 | 1.010 | 0.345 | 0.272 | 0.331 | 0.284 | 0.233 | 0.701 | 0.245 |
| **SDMA** | 1.220 | 0.150 | 3.650 | 1.150 | 0.150 | 1.150 | 0.774 | 0.901 | 1.320 | 1.440 | 1.780 | 2.540 | 3.300 | 1.710 | 1.680 | 1.370 | 3.340 | 3.870 | 2.190 | 1.110 |
| **Spermidine** | 0.124 | 0.311 | 0.156 | 0.093 | 0.058 | 0.060 | 0.437 | 0.132 | 0.060 | 0.249 | 0.108 | 0.199 | 0.161 | 0.065 | 0.044 | 0.049 | 0.089 | 0.041 | 0.103 | 0.201 |
| **Spermine** | 0.041 | 0.454 | 0.051 | 0.079 | 0.024 | 0.034 | 0.218 | 0.030 | 0.010 | 0.108 | 0.034 | 0.048 | 0.028 | 0.041 | 0.019 | 0.020 | 0.077 | 0.029 | 0.061 | 0.104 |
| **T4 OH Pro** | 8.040 | 6.550 | 9.250 | 36.000 | 10.200 | 6.690 | 9.760 | 11.600 | 18.800 | 13.400 | 5.650 | 161.00 | 6.850 | 12.700 | 7.360 | 5.230 | 11.800 | 5.190 | 24.600 | 4.360 |
| **Taurine** | 61.600 | 47.500 | 48.100 | 19.800 | 27.700 | 34.400 | 136.00 | 12.100 | 21.900 | 40.500 | 11.800 | 24.100 | 19.100 | 12.400 | 35.100 | 11.900 | 31.200 | 9.370 | 41.200 | 59.200 |
| **Total DMA** | 2.050 | 0.585 | 3.680 | 1.870 | 0.731 | 1.530 | 1.300 | 1.220 | 2.050 | 2.050 | 2.270 | 5.120 | 4.200 | 2.720 | 1.960 | 1.820 | 4.300 | 4.420 | 3.790 | 1.780 |
| **SM OH C14:1** | 3.052 | 3.027 | 1.895 | 3.372 | 1.232 | 2.229 | 2.219 | 1.520 | 1.262 | 2.860 | 2.290 | 3.114 | 3.159 | 2.455 | 2.112 | 2.186 | 2.986 | 2.252 | 1.257 | 2.093 |
| **SM OH C16:1** | 1.242 | 0.983 | 0.702 | 1.066 | 0.566 | 0.825 | 0.822 | 0.508 | 0.400 | 0.906 | 0.862 | 0.899 | 1.346 | 0.911 | 0.970 | 0.724 | 0.803 | 0.699 | 0.386 | 0.753 |
| **SM OH C22:1** | 0.614 | 0.411 | 0.431 | 0.659 | 0.286 | 0.507 | 0.546 | 0.351 | 0.265 | 0.539 | 0.494 | 0.339 | 0.950 | 0.586 | 0.436 | 0.436 | 0.350 | 0.499 | 0.194 | 0.545 |
| **SM OH C22:2** | 0.811 | 0.612 | 0.521 | 0.551 | 0.350 | 0.728 | 0.545 | 0.281 | 0.236 | 0.555 | 0.693 | 0.581 | 0.959 | 0.687 | 0.592 | 0.412 | 0.513 | 0.615 | 0.229 | 0.437 |
| **SM OH C24:1** | 0.076 | 0.018 | 0.057 | 0.061 | 0.008 | 0.033 | 0.086 | 0.051 | 0.025 | 0.037 | 0.040 | 0.080 | 0.070 | 0.061 | 0.032 | 0.041 | 0.044 | 0.045 | 0.023 | 0.035 |
| **SM C16:0** | 49.881 | 46.158 | 36.919 | 65.917 | 29.959 | 42.706 | 54.322 | 45.173 | 32.254 | 45.278 | 53.135 | 65.025 | 60.646 | 43.708 | 44.344 | 43.623 | 84.369 | 45.828 | 23.762 | 40.328 |
| **SM C16:1** | 6.007 | 6.312 | 4.967 | 7.828 | 3.863 | 6.832 | 6.698 | 4.912 | 4.799 | 8.140 | 7.427 | 9.566 | 10.533 | 6.283 | 6.248 | 5.930 | 10.400 | 6.637 | 2.341 | 4.956 |
| **SM C18:0** | 6.278 | 4.901 | 3.097 | 5.146 | 3.586 | 4.042 | 4.386 | 2.351 | 1.825 | 6.121 | 5.658 | 2.328 | 8.209 | 4.558 | 6.372 | 3.567 | 3.666 | 3.442 | 1.413 | 3.497 |
| **SM C18:1** | 2.688 | 2.184 | 1.322 | 2.107 | 1.622 | 2.112 | 1.856 | 1.076 | 1.136 | 3.219 | 2.768 | 1.420 | 4.639 | 2.178 | 3.151 | 1.774 | 1.842 | 1.947 | 0.591 | 1.583 |
| **SM C20:2** | 0.109 | 0.092 | 0.060 | 0.121 | 0.068 | 0.081 | 0.096 | 0.037 | 0.034 | 0.136 | 0.079 | 0.084 | 0.189 | 0.092 | 0.084 | 0.081 | 0.081 | 0.087 | 0.041 | 0.038 |
| **SM C22:3** | 0.005 | 0.045 | 0.044 | 0.150 | 0.014 | 0.005 | 0.044 | 0.006 | 0.033 | 0.005 | 0.036 | 0.054 | 0.005 | 0.005 | 0.005 | 0.015 | 0.039 | 0.032 | 0.005 | 0.005 |
| **SM C24:0** | 0.728 | 0.820 | 0.692 | 1.155 | 0.489 | 0.705 | 0.892 | 0.811 | 0.605 | 0.860 | 0.956 | 1.028 | 1.364 | 0.809 | 0.776 | 0.594 | 0.841 | 0.777 | 0.525 | 1.160 |
| **SM C24:1** | 3.269 | 3.244 | 3.025 | 3.294 | 2.030 | 2.800 | 3.004 | 2.477 | 2.491 | 3.166 | 4.045 | 3.913 | 3.571 | 2.449 | 2.476 | 2.409 | 3.726 | 3.823 | 1.756 | 2.590 |
| **SM C26:1** | 0.013 | 0.021 | 0.012 | 0.011 | 0.005 | 0.025 | 0.004 | 0.018 | 0.016 | 0.021 | 0.023 | 0.007 | 0.020 | 0.036 | 0.018 | 0.014 | 0.001 | 0.014 | 0.001 | 0.017 |
| **C0** | 71.405 | 32.324 | 58.249 | 270.49 | 17.512 | 25.005 | 115.62 | 55.907 | 110.21 | 17.337 | 52.166 | 112.81 | 75.522 | 49.983 | 59.642 | 69.854 | 101.77 | 66.351 | 110.63 | 28.718 |
| **C2** | 11.424 | 2.782 | 5.094 | 34.921 | 1.529 | 2.523 | 9.413 | 3.741 | 9.223 | 2.836 | 6.984 | 22.734 | 6.993 | 3.711 | 5.619 | 7.352 | 21.904 | 8.323 | 15.557 | 2.299 |
| **C4** | 1.058 | 0.645 | 0.622 | 7.272 | 0.227 | 0.632 | 0.767 | 0.263 | 0.441 | 0.272 | 0.524 | 1.371 | 0.765 | 0.520 | 0.699 | 0.374 | 1.543 | 0.949 | 1.686 | 0.784 |

|  | **10 features** | | **20 features** | | **30 features** | |
| --- | --- | --- | --- | --- | --- | --- |
| **METABOLITES** | **min Dev** | **fixed λ** | **min Dev** | **fixed λ** | **min Dev** | **fixed λ** |
| **PC aa C42:6** | **-0.763** | **-0.213** | **-0.672** | **-2.083** | **-0.557** | **-0.466** |
| **PC aa C40:6** | - | - | - | - | -0.380 | -0.005 |
| **PC ae C42:1** | - | - | - | -1.009 | - | - |
| **lysoPC a C24:0** | -0.498 | -0.223 | - | -0.622 | -0.233 | -0.241 |
| **lysoPC a C20:4** | - | - | - | - | -0.188 | -0.025 |
| **SM OH C16:1** | - | - | - | -1.137 | - | - |
| **SM C24:1** | - | - | - | 0.263 | -0.182 | - |
| **SM C22:3** | - | - | - | - | - | -0.167 |
| **SM C24:0** | - | - | - | - | - | -0.030 |
| **PC ae C42:5** | - | - | - | - | -0.160 | - |
| **PC aa C42:2** | -1.103 | - | -0.136 | - | -0.149 | - |
| **PC aa C34:4** | -1.333 | - | - | -0.191 | - | - |
| **Met** | - | - | - | - | -0.105 | -0.082 |
| **PC ae C30:2** | - | - | - | -0.565 | -0.073 | -0.173 |
| **PC aa C36:6** | - | - | - | - | 0.013 | - |
| **PC aa C42:5** | - | - | - | - | 0.063 | 0.271 |
| **PC aa C36:3** | **2.280** | **0.338** | **0.442** | **1.931** | **0.178** | **0.479** |
| **Pro** | - | - | 0.652 | 1.946 | 0.198 | - |
| **PC aa C34:3** | - | - | 0.262 | - | 0.582 | - |
| **PC aa C42:1** | - | - | 1.135 | 2.824 | 0.653 | 0.716 |
| **Tyr** | **3.151** | **0.061** | **0.021** | **1.075** | **0.751** | **0.126** |
| **PC ae C30:1** | - | - | 0.305 | 1.151 | 0.820 | 0.300 |
| **Creatinine** | - | - | 0.377 | 1.489 | 1.623 | 0.253 |
| **Performance** | Dev=4.02 | Dev=23.77 | Dev=8.69 | Dev=24.98 | Dev=9.15 | Dev=25.62 |

**Table S5** - Coefficient values of the logistic regression models for the first 10, 20 and 30 metabolites, computed according the two strategies (minimal deviance and estimated λ). The coefficients of the metabolites which are common to all models are in bold. The bottom row reports values of deviance of the obtained models.

|  | **10 features** | | **20 features** | | **30 features** | |
| --- | --- | --- | --- | --- | --- | --- |
| **FEATURES** | **min Dev** | **fixed λ** | **min Dev** | **fixed λ** | **min Dev** | **fixed λ** |
| **P02790** | - | - | -0.416 | -0.227 | -1.630 | -0.354 |
| **lysoPC a C24:0** | **-1.175** | **-0.641** | **-0.993** | **-0.372** | **-1.251** | **-0.628** |
| **PC aa C42:6** | - | - | -0.801 | -0.395 | -0.186 | -0.579 |
| **P02745** | **-1.087** | **-0.829** | **-0.187** | **-0.289** | **-0.774** | **-0.497** |
| **P20851** | -0.485 | - | - | - | - | - |
| **lysoPC a C17:0** | - | - | - | - | -0.306 | -0.105 |
| **SM OH C16:1** | - | -0.096 | -0.335 | -0.050 | - | -0.487 |
| **P02746** | - | - | -0.235 | -0.091 | -0.249 | -0.347 |
| **PC aa C42:2** | -0.064 | - | - | - | -0.044 | - |
| **PC aa C34:3** | - | - | - | 0.115 | 0.012 | - |
| **PC ae C30:1** | - | - | - | - | 0.017 | 0.346 |
| **O75882** | 0.389 | 0.239 | - | 0.086 | 0.212 | 0.472 |
| **Pro** | - | - | 0.054 | - | 0.238 | 0.169 |
| **P06276** | - | - | - | - | 0.240 | - |
| **P06727** | - | - | - | - | - | 0.245 |
| **P19823** | - | - | 0.701 | 0.357 | 0.256 | 0.913 |
| **P05543** | 0.093 | 0.301 | 0.011 | 0.108 | - | 0.327 |
| **PC ae C42:1** | - | - | -0.286 | - | 0.283 | - |
| **Tyr** | - | - | - | - | - | 0.309 |
| **P01034** | - | - | - | - | 0.909 | 0.895 |
| **PC aa C36:3** | - | - | 0.628 | 0.343 | 1.184 | 0.888 |
| **Performance** | Dev=10.6 | Dev=33.89 | Dev=8.66 | Dev=21.52 | Dev=3.96 | Dev=16.21 |

**Table S6** - Coefficient values of the logistic regression models for integration of metabolomics and proteomics built on the first 10, 20 and 30 features and computed according the two strategies (minimal deviance and estimated λ). The coefficients of the features which are in common to all models are in bold. The bottom row reports values of deviance of the obtained models.

|  | **10 features** | | **20 features** | | **30 features** | |
| --- | --- | --- | --- | --- | --- | --- |
| **FEATURES** | **min Dev** | **fixed λ** | **min Dev** | **fixed λ** | **min Dev** | **fixed λ** |
| **P02745** | **-1.140** | **-0.664** | **-0.410** | **-0.805** | **-0.374** | **-0.450** |
| **PC ae C34 3** | - | - | - | - | -0.235 | - |
| **Mean Arterial Pressure** | -0.580 | -0.148 |  | -0.255 | -0.221 | - |
| **PC aa C34 3** | **-0.315** | **-0.325** | **-0.279** | **-0.271** | **-0.187** | **-0.027** |
| **PaCO2** | - | - | -0.192 | -0.539 | -0.138 | -0.118 |
| **P02790** | **-0.273** | **-0.306** | **-0.383** | **-0.229** | **-0.117** | **-0.135** |
| **ScvO2** | - | - | -0.082 | -0.067 | - | - |
| **Serum bilirubin** | - | 0.029 |  |  | - | - |
| **FiO2** | - | - | 0.137 | 0.291 | - | 0.023 |
| **O75882** | - | - | - | - | 0.150 |  |
| **P20851** | - | - | - | - | 0.169 |  |
| **PC ae C44 4** | - | - | - | - | 0.211 | 0.149 |
| **PEEP** | 0.738 | 0.299 | - | 0.439 | 0.267 | 0.054 |
| **Central Venous Pressure** | - | - | - | - | 0.372 | - |
| **Heart Rate** | 0.124 | - | - | - | - | - |
| **P06276** | - | - | 0.260 | - | - | - |
| **PC ae C44 4** | - | - | 0.379 | 0.572 | - | - |
| **Urine Output** | 0.414 | - | 0.138 | 0.242 | - | - |
| **Serum creatinine** | - | - | 0.432 | 0.423 | - | 0.033 |
| **Performance** | Dev=10.22 | Dev=27.08 | Dev=11.65 | Dev=32.83 | Dev=11.75 | Dev=30.04 |

**Table S7** - Coefficient values of the logistic regression models for integration of omics data with clinical parameters built on the first 10, 20 and 30 features and computed according the two strategies (minimal deviance and estimated λ). The coefficients of the features which are in common to all models are in bold. The bottom row reports values of deviance of the obtained models.
